# Micro-scale patchiness enhances trophic transfer efficiency and potential plankton biodiversity

**DOI:** 10.1038/s41598-019-53592-6

**Published:** 2019-11-21

**Authors:** Anupam Priyadarshi, S. Lan Smith, Sandip Mandal, Mamoru Tanaka, Hidekatsu Yamazaki

**Affiliations:** 1 Department of Mathematics, Institute of Science, Banaras Hindu University, Varanasi, 221005 India; 2 Earth SURFACE System Research Center, Research Institute for Global Change (RIGC), Japan Agency for Marine-Earth Science and Technology, 3173-25 Showa-machi, Yokohama, 236-0001 Japan; 3 Department of Ocean Sciences, Tokyo University of Marine Science and Technology, Minato-ku, Tokyo 108-8477 Japan; 4 Translational Global Health and Policy Research Cell, Indian Council of Medical Research, New Delhi, 110001 India

**Keywords:** Biogeochemistry, Marine biology

## Abstract

Rather than spatial means of biomass, observed overlap in the intermittent spatial distributions of aquatic predators and prey is known to be more important for determining the flow of nutrients and energy up the food chain. A few previous studies have separately suggested that such intermittency enhances phytoplankton growth and trophic transfer to sustain zooplankton and ultimately fisheries. Recent observations have revealed that phytoplankton distributions display consistently high degrees of *mm* scale patchiness, increasing along a gradient from estuarine to open ocean waters. Using a generalized framework of plankton ecosystem models with different trophic configurations, each accounting for this intermittency, we show that it consistently enhances trophic transfer efficiency (*TE*), i.e. the transfer of energy up the food chain, and expands the model stability domain. Our results provide a new explanation for observation-based estimates of unexpectedly high *TE* in the vast oligotrophic ocean and suggest that by enhancing the viable trait space, micro-scale variability may potentially sustain plankton biodiversity.

## Introduction

Environmental heterogeneity plays major roles in determining population dynamics, community structure, ecosystem productivity, and biodiversity patterns^1–3^. It has also been found to promote biodiversity and coexistence in many ecosystems^3–5^ which tends to enhance productivity and related energy and mass fluxes^6^. Thus, it is important to understand how intermittency of habitat conditions impacts biodiversity and ecosystem functions. Particularly for plankton, although many diversity-sustaining processes have been identified, a clear explanation remains lacking for why the number of coexisting species typically far exceeds the number of limiting resources, i.e. the long-standing ‘Paradox of the Plankton’^7^.

Recent observations have revealed ubiquitous intermittency in phytoplankton distributions at the micro (*mm*) scale^8–11^, and a few recent modelling studies have suggested that this micro-scale variability impacts plankton ecosystem dynamics and biodiversity^12–14^. Modelling is an essential tool for studying complex food webs, by linking the observed ecological patterns with experimental findings to understand mechanisms and make future predictions. However, so far almost all ecosystem models have been developed based on the mean-field approach, i.e. assuming well mixed environmental conditions within each discretely resolved grid cell. Although this is reasonable for examining patterns at the meso- (*km*) to global scales, it is not realistic for plankton, which experience micro-scale variability in aquatic environments where predator-prey overlap can substantially enhance trophic transfer^15,16^. Similar to a recent study that addressed variability at the scale of ocean fronts^16^, we have recently used the Reynolds decomposition and a truncated Taylor series to develop ‘moment closure’ models accounting for micro-scale variability in the distributions of Nutrients and Phytoplankton (NP closure model)^12^, and also Zooplankton (NPZ closure model)^14^. Compared to conventional ecosystem models based on the mean-field approach, these closure models yield considerably different dynamics.

Using a generalized plankton ecosystem modelling framework, including models of differing trophic complexity and with different grazing functional responses, we investigate how micro-scale variability affects plankton biodiversity and ecosystem function. Specifically, we apply the closure modelling approach to test the functional forms often assumed in ecosystem models against observed micro-scale intermittency as quantified by the *coefficient of variation (CV)* (ratio of standard deviation to mean) of the micro-scale fluorescence field, which is a proxy for phytoplankton biomass. With both saturating and non-saturating grazing functions and in all model configurations considered, we find that micro-scale variability consistently supports the highest trophic level present, i.e. enhances trophic transfer efficiency (*TE*), and expands the model stability domain, potentially sustaining biodiversity by allowing species with a wider range of trait values to coexist.

## Materials and Methods

### Observations

A cylinderically shaped free-fall microstructure profiler “Turbulence Ocean Microstructure Acquisition Profiler-Laser” (TurboMAP-L^8^) which is 2 m in length, 0.12 m in diameter and 30 kg in air is used to observe the *CV* of fluorescence in Tokyo Bay, Japan. TurboMAP-L carries physical and biological sensors on its head (front end): a millimetre (*mm*) scale resolution laser fluorescence sensor (256 Hz), a light-emitting diode fluorescence/turbidity probe (256 Hz), two turbulent shear probes (512 Hz), a FP07 fast temperature probe (512 Hz), a CTD sensor (64 Hz), and a XYZ 3-directional accelerometer (256 Hz). Each sensor measures undisturbed environments as TurboMAP-L falls freely downward into the ocean. Profiling speed was about 0.5 m s^−1^ in this study. The laser fluorescence sensor, whose sampling volume is 32 μL, captures *in situ* variability of the fluorescence field with an effective resolution of 2 mm^8^.

We calibrated the high resolution laser fluorescence sensor with water samples collected using a Rosette sampler. Phytoplankton cells were filtered using Whatman GF/B filters with a particle retention of 1 μm just after the water sampler was recovered to the ship. Filters were stored at −4 °C, shaded from light. Within a week after the cruise, we measured chlorophyll-*a* concentrations using the Turner Designs fluorometer. These *in situ* chlorophyll-*a* concentrations are average values over the 1 m height of the sampling bottles. A linear calibration equation was obtained by comparing the corresponding spatial averages of the high resolution fluorescence profiles.

**Figure 1:**
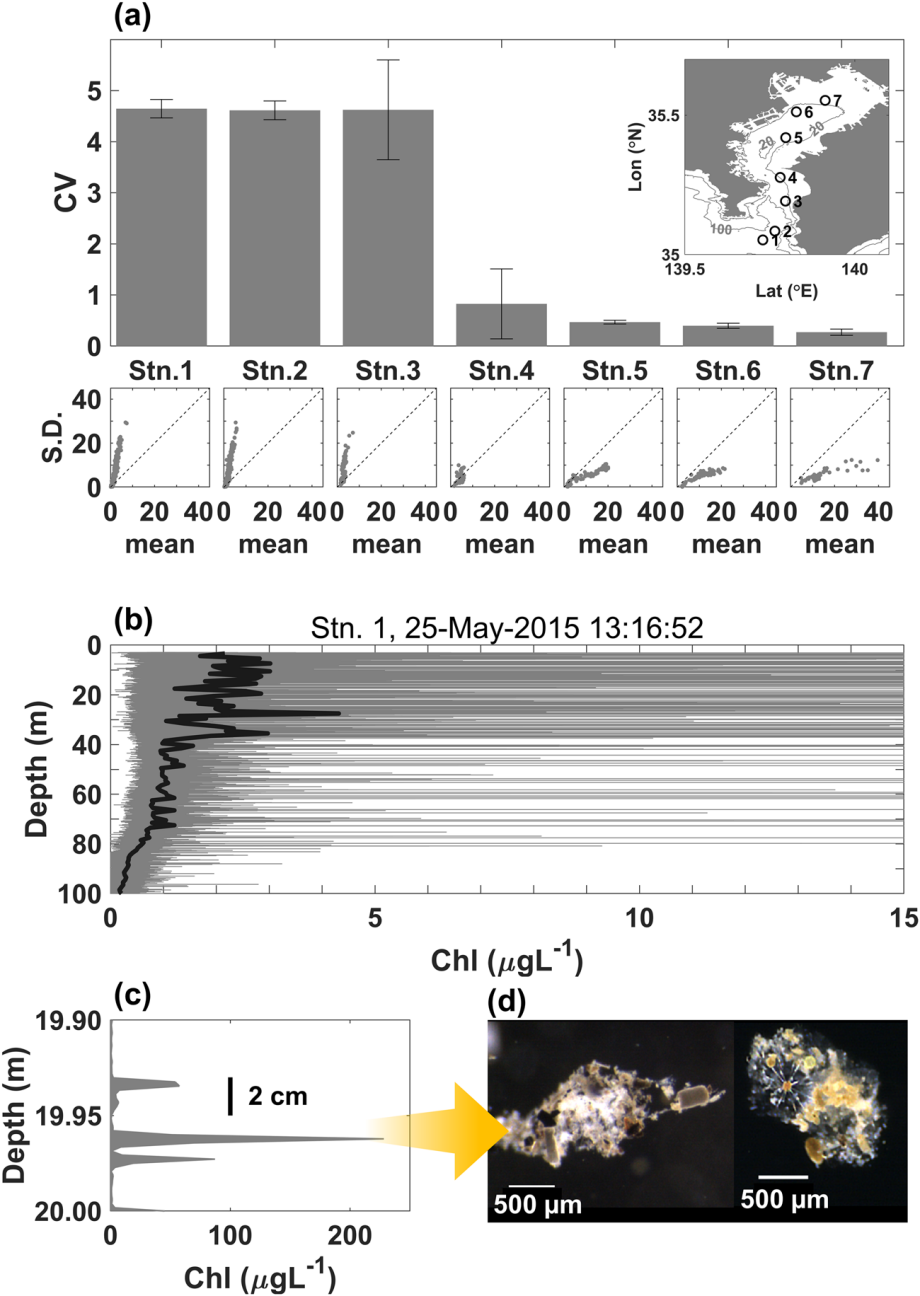
(**a**) Observation stations (open, numbered circles) in Tokyo bay (locations and depths given in Table 1), *CV* estimates (bar heights, with error bars indicating 99% confidence intervals) and corresponding scatter plots of observed standard deviation vs. mean of fluorescence data from each station (dotted lines showing one-to-one relationships). (**b**) Raw data (thin grey lines) and 1 m average values (thick black lines) from a vertical fluorescence profile acquired at stn. 1 by the laser sensor, which was calibrated with *in situ* water samples to obtain values in μg chl L^−1^. (**c**) A subset of peaks from the same profile around 20 m depth. Vertical thick black bar indicates a scale bar of 2 cm height. (**d**) Images of typical aggregates.

A field campaign was conducted on May 24 to 25^th^, 2015 in Tokyo Bay, Japan (Fig. 1, Table 1), which is a semi-enclosed bay connected with the Pacific Ocean by the Sagami-nada Sea. While the outer part of the bay is affected by the Kuroshio Current (the western North Pacific boundary current), the inner part of the bay is affected by freshwater inflow of about 13 billion tons annually throughout the bay^17^. This typically induces horizontal gradients of temperature and salinity, as well as of chemical (e.g. nutrients) and biological (e.g. phytoplankton abundance and community structure) characteristics^18,19^.

**Table 1:** Information about the observation stations, TurboMAP-L deployments, and *CV* of fluorescence for each station.

Station name	Long. [deg. E]	Lat. [deg. N]	Depth [m]	TurboMAP-L			
				Day	Start time	Profile depth [m]	*CV* [ND]
1	139.730	35.052	600	24^th^	08:35	120	4.6
2	139.765	35.084	500	24^th^	09:50	110	4.6
3	139.796	35.192	24	24^th^	11:27	22	4.6
4	139.781	35.277	22	24^th^	12:51	17	0.8
5	139.797	35.420	28	25^th^	09:57	23	0.5
6	139.828	35.512	26	25^th^	11:13	18	0.4
7	139.913	35.553	18	25^th^	13:12	15	0.3

### Quantifying intermittency of phytoplankton distributions

High resolution (*mm* scale) fluorescence profiles were observed using a laser florescence probe mounted on a microstructure profiler TurboMAP-L^8^ as explained in Results. To evaluate variability (or unevenness) of phytoplankton field, we calculated the *Coefficient of Variation* (*CV*) of the observed fluorescence:

1 $$CV=\frac{{\rm{standard}}\,{\rm{deviation}}}{{\rm{mean}}}$$

Our models represent the *CV* as the ratio of the fluctuating component (standard deviation) to the mean-field of phytoplankton biomass (See Appendix).

### Trophic transfer efficiency

We calculated the trophic transfer efficiency (*TE*) from phytoplankton to zooplankton as the ratio of primary to secondary production^20,21^:

2 $$TE=\frac{{\rm{Zooplankton}}\,{\rm{production}}\,{(\mathrm{gCm}}^{-{\rm{3}}}\,{{\rm{d}}}^{-{\rm{1}}})}{{\rm{Phytoplankton}}\,{\rm{production}}\,{(\mathrm{gCm}}^{-{\rm{3}}}\,{{\rm{d}}}^{-{\rm{1}}})}$$

where production is the rate in terms of carbon biomass. We calculated *TE* using production rates (mol N m^−3^ d^−1^) from our nitrogen-based models, assuming for the sake of simplicity and generality a constant Redfield C:N ratio of 6.7 (mol mol^−1^)^22^ for all biomass.

### Methods and model formulation

We examined the impact of variability in the following plankton models:

(i) NP (Nutrient-Phytoplankton) model
(ii) NPZ (Nutrient-Phytoplankton-Zooplankton) model with different zooplankton grazing functional: Linear, Holling Type II and Holling type III
(iii) NPZD (Nutrient-Phytoplankton-Zooplankton-Detritus) model with Linear, Holling Type II and Holling type III Z-grazing functional response.

The moment closure form of each model, which accounts for micro-scale variability, may be obtained from the general NPZD closure model developed below.

### Conventional NPZD model

Based on the mean-field approach (first central moment), the ecological interactions of nutrient (*N*), phytoplankton (*P*), zooplankton (Z) and detritus (D) can be written in generalized form as:

3 $$\frac{dN}{dt}=-{\nu }_{max}\,\frac{N}{K+N}P+{M}_{ZN}\,Z+{\gamma }_{M}\,D$$

4 $$\frac{dP}{dt}={\nu }_{max}\,{F}_{N}(N)\,P-{M}_{P}\,P-{G}_{P}(R,\hat{\mu },P)\,Z$$

5 $$\frac{dZ}{dt}=(1-\gamma )\,{G}_{P}(R,\hat{\mu },P)\,Z-{M}_{ZN}\,Z-{M}_{ZD}\,Z$$

6 $$\frac{dD}{dt}={M}_{P}P+\gamma {G}_{P}(R,\hat{\mu },P)\,Z+{M}_{ZD}\,Z-{\gamma }_{M}\,D$$

The growth of phytoplankton depends on the light intensity and nutrient (*N*) availability ${F}_{N}(N)=\frac{N}{K+N}$. Here, linear mortality rates are assumed for both phytoplankton and zooplankton. The other parameters have the same meaning and their details are stated in Table S1 (Appendix). In both the NPZ and NPZD models, the following three zooplankton grazing functional responses are studied:

(i) Linear $${G}_{P}(R,\hat{\mu },P)=RP$$

(ii) Holling type II ${G}_{P}(R,\hat{\mu },P)=\frac{RP}{\hat{\mu }+P}$

(iii) Holling type III ${G}_{P}(R,\hat{\mu },P)=\frac{R{P}^{2}}{{\hat{\mu }}^{2}+{P}^{2}}$

Here parameter *R* is the maximum rate of zooplankton grazing on phytoplankton, and $\hat{\mu }$ is the half-saturation constant of the z-grazing functional response. The above three functional responses are widely used in planktonic modelling^2,4,12,14,16,23–25^.

### Closure models

Closure models are derived using the Reynolds decomposition as is widely used in turbulence studies. Variables as functions of time (*t*) and space (*s*) are each decomposed into mean and fluctuating components as

$$N(s,t)={N}_{0}(s,t)+N^{\prime} (s,t),\,P(s,t)={P}_{0}(s,t)+P^{\prime} (s,t)$$

7 $$Z(s,t)={Z}_{0}(s,t)+Z^{\prime} (s,t),\,D(s,t)={D}_{0}(s,t)+D^{\prime} (s,t)$$

Here, the fluctuating components may be either positive or negative. Observed phytoplankton profiles reveal that most of these fluctuating components are small relative to the mean value, with few components having values greater than the mean value. According to field measurements based on a laser fluorescence probe, *P*′ follows a *Gumbel extreme value* probability distribution^8,9^. A large fraction of signals from this probability distribution appears below the mean. Therefore, we use the Taylor expansion around the mean of each variable and retain the terms up to second order only to approximate the temporal variation of variance and covariance in the closure model. Observations of microscale profiles of phytoplankton using different instruments (such as Seapoint fluorometer, Light Emitting Diode (LED) sensor, Laser sensor), suggest that at a particular time, the depth-average of the fluctuating components is zero^12^. Therefore, ($\langle N^{\prime} (s)\rangle =\langle P^{\prime} (s)\rangle =\langle Z^{\prime} (s)\rangle =\langle D^{\prime} (s)\rangle =0$), while the temporal average can be nonzero, which also implies $\langle N(s)\rangle ={N}_{0}(s),\,\langle P(s)\rangle ={P}_{0}(s),\,\langle Z(s)\rangle ={Z}_{0}(s)\,{\rm{and}}\,\langle D(s)\rangle \,=\,{D}_{0}(s)$. With these assumptions, we can derive equations for the temporal variation of mean and fluctuating components for all variables.

### Generalized closure model framework

By assuming $f={F}_{N}(N)P$ as nutrient uptake by phytoplankton and ${g}_{p}={G}_{P}(R,\hat{\mu },P)Z$ as the zooplankton grazing response function, a generalized closure model framework has been developed (for details, see Appendix).

## Results

Horizontal gradients were observed for chlorophyll-*a*, salinity, and temperature along the transect (Fig. S1 Appendix) in the upper layer (above 30 m depth). Chlorophyll-*a* concentrations were >10 *μg*L^−1^ for the upper 10 m layer at Stns. 5 to 7 and reached 20 μg L^−1^ at 5 m depth at Stn. 7 (Fig. S1a). On the other hand, chlorophyll-*a* values were <3 *μg*L^−1^ at Stns. 1 to 3 (Fig. S1a). Stn. 4 had intermediate values ≈5 μg L^−1^. Lower salinity and higher temperature values were observed in the inner bay: salinity and temperature at Stn. 1 were ≈34 PSU and ≈18 °C respectively, while these were ≈30 PSU and ≈20 °C at Stn. 7 (Fig. S1b,c). For deep layers (<30 m depth; only at Stn. 1 and 2), chlorophyll-*a* concentrations were <1 *μg*L^−1^ on average, salinity was almost homogeneous ≈34.5 PSU, and temperature was ≈16 °C at 30 m depth and decreased with depth at a rate of about −0.04 °C m^−1^ to 100 m depth. By water mass analysis, we found that Stns. 1 to 3 had similar water mass properties (relatively saline and cold), and the water became less saline and warmer going towards Stns. 4 through Stn. 7 (Fig. S2 Appendix). The information about the observation stations, TurboMAP-L deployments, and *CV* of fluorescence for each station is listed in Table 1.

A typical profile near the entrance to Tokyo Bay (Fig. 1a) reveals high intermittency (thin grey lines), with numerous sub-centimeter local peaks of high chlorophyll. The chlorophyll peaks reach 200 $\mu g\,{\rm{chlorophyll}}\,a\,{{\rm{L}}}^{-1}$(Fig. 1c), while background values are $< 3\,\mu g\,{\rm{chlorophyll}}\,a\,{{\rm{L}}}^{-1}$ (thick black line in Fig. 1b). Water mass analysis reveals similar conditions (relatively salty and cold) near the mouth of the bay, with a tendency for warmer waters to be less saline (Fig. S2 Appendix).

**Figure 2:**
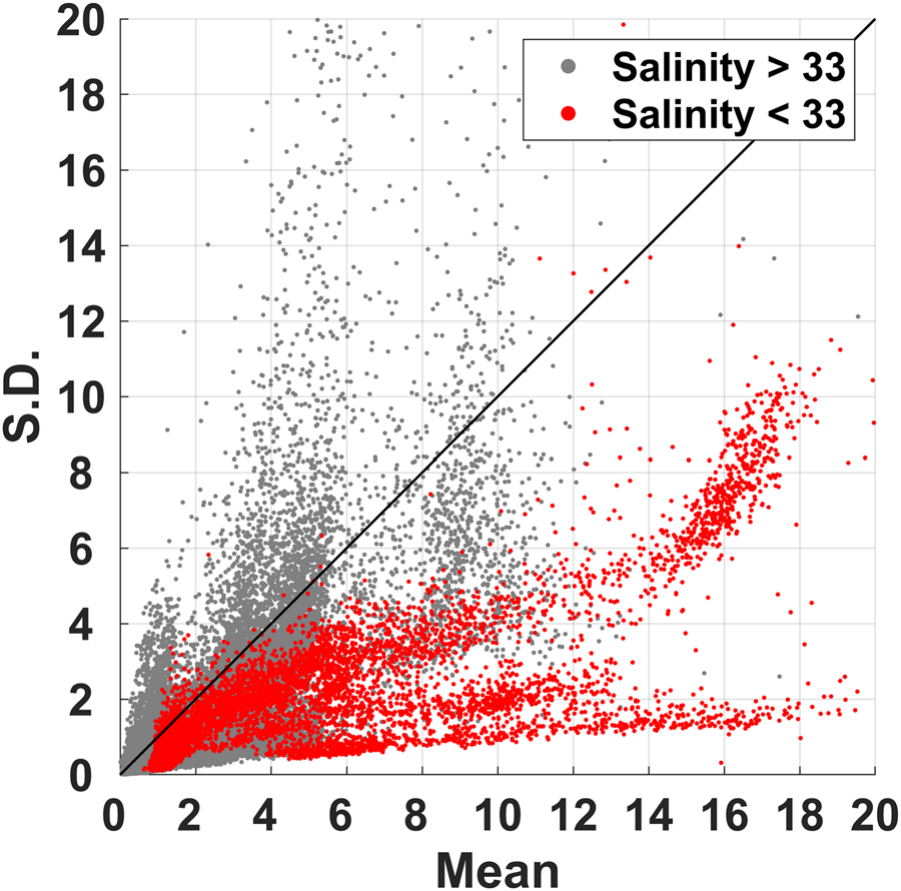
Mean-standard deviation diagram of high-resolution fluorescence acquired from 33 cruises. Gray dots denote data acquired from high salinity water (>33 PSU), and red dots denote those acquired from low salinity water (<33 PSU). The number of samples is N = 56330.

*CV* values were higher (lower) in the outer (inner) bay (Fig. 1a), reaching 4*.6* at the outermost stn. 1 but only *0.3* at the innermost stn. 7. Data from 33 field campaigns reveal a strong positive relationship between *CV* and salinity (Fig. 2), with *CV* values **>**4 in high salinity (mostly open ocean) waters, and <1 in the low salinity estuarine waters of the inner bay.

**Figure 3:**
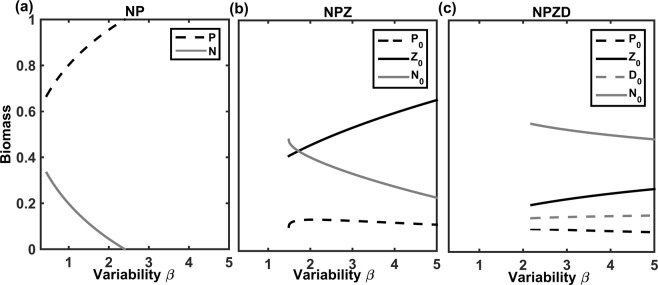
Increasing normalised micro-scale variability, β, consistently enhances the highest trophic level present in each model, namely phytoplankton biomass in (**a**) the NP model, and zooplankton biomass in the (**b**) NPZ and (**c**) NPZD models.

**Figure 4:**
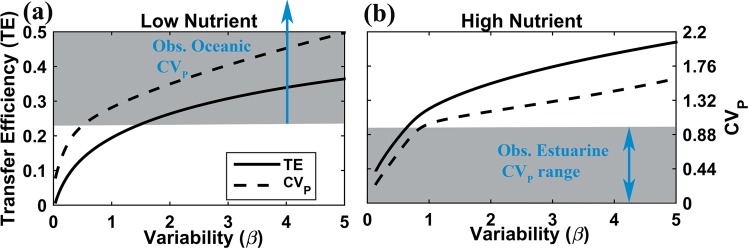
Trophic transfer efficiency, *TE*, from phytoplankton to zooplankton and the observed *CV* value both consistently increase with total normalised micro-scale variability, β, under (**a**) low and (**b**) high nutrient conditions. Lower values of β give *TE* values ~0.1, in agreement with most typical estimates^20^ and *CV* values consistent with observations from low salinity estuarine waters. Higher values of β give *TE* values ~ 0.2–0.4, consistent with estimates from the open ocean^27,28^.

In all model configurations examined, micro-scale variability enhances trophic transfer, supporting higher trophic levels (Fig. 3). Modelled *TE* increased consistently with total micro-scale variability, quantified as β, the ratio of total summed variances and covariances to the square of the sum of all mean components (Fig. 4). Although we have observations of *CV* only, a consistently strong and positive relationship exists between *CV* and micro-scale variability, β (Fig. S13 in Appendix, see also^14^). The highest modelled values of *TE* were obtained for β values corresponding to observed phytoplankton *CV* from open ocean waters (Fig. 4).

**Figure 5:**
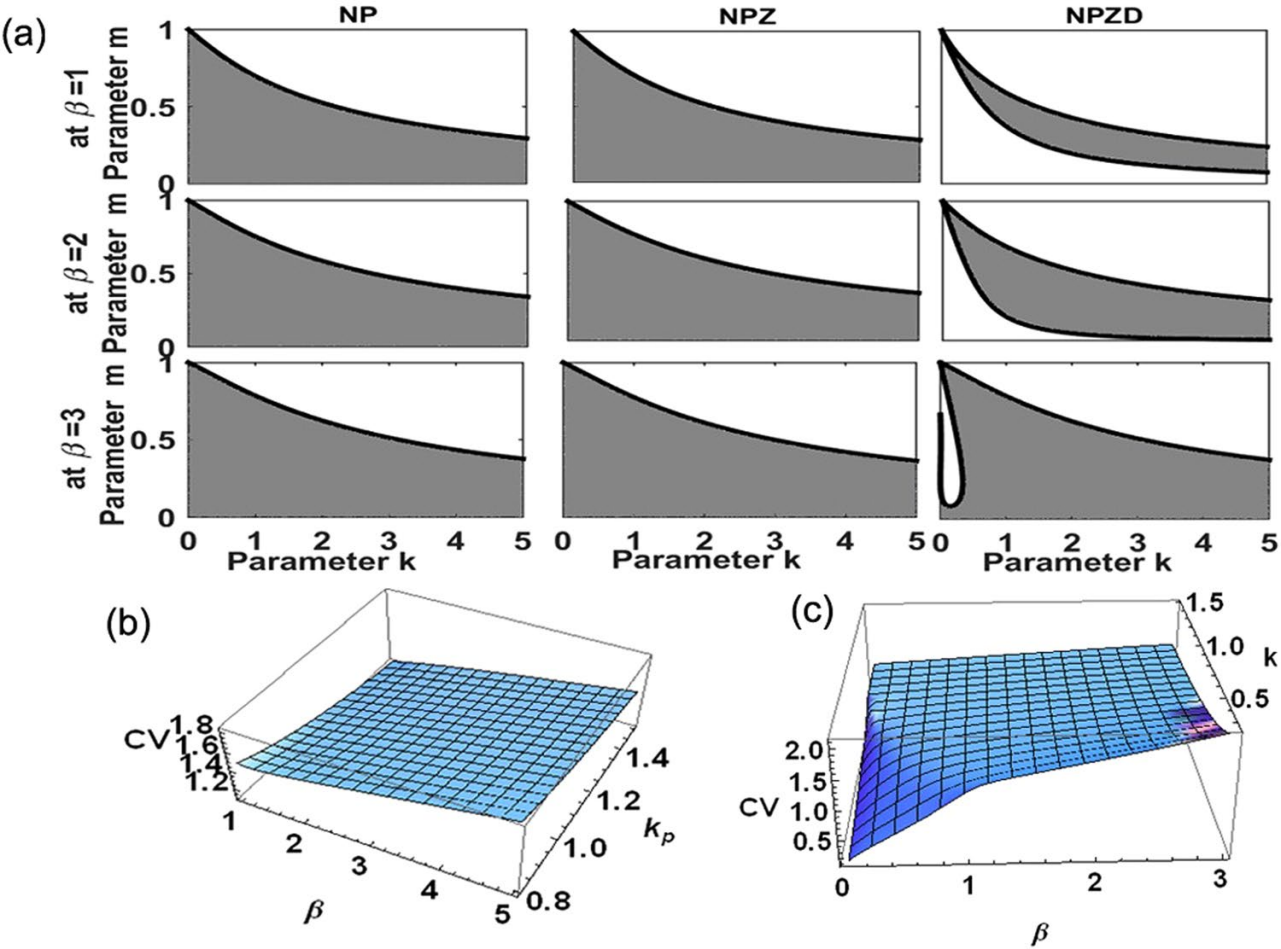
(**a**) The stability domain, i.e. the range of parameter values allowing stable solutions, expands consistently with increasing micro-scale variability, β, for all plankton closure models examined. (**b**) Stable solutions are possible with *CV* > 1 for all plankton models using different grazing functional response, and (**c**) only the Holling Type-II grazing functional response allows stable solutions with *CV* < 1.

Micro-scale variability also enhances the stability domain of all plankton ecosystem models examined, with different levels of trophic complexity (Fig. 5a). Only models using the Holling type-II grazing functional response were able to produce stable solutions over the full observed range of *CV* (0.1–5.4) for phytoplankton. However, CV < 1 of phytoplankton is obtained only for a narrow range of the parameter domain (Fig. 5b,c).

## Discussion

Our finding that micro-scale heterogeneity consistently enhances the biomass of the highest trophic level present in each of our model configurations agrees well with independent observations. Detailed observations of the frequency and intensity of spatio-temporal aggregation have revealed it to be more important than mean-field biomass for determining trophic interactions of marine organisms ranging from plankton to dolphins^26^. A recent meta-analysis of observed predator and prey distributions reported enhancement of trophic transfer by predator-prey overlap for aquatic organisms ranging from phytoplankton to whales^15^. Although that observation-based study considered larger scales of variability (~1 to 10 m) compared to the *mm*-scale variability considered herein, it also found that increasing the resolution of observations (i.e., considering finer spatial scales) enhanced the positive effect of predator-prey overlap on trophic transfer and consequently the degree to which the mean-field approximation under-estimates productivity at higher trophic levels. This implies that micro-scale variability may control the efficiency of trophic transfer to larger plankton and thereby the ability of plankton ecosystems to support fish stocks and the important ecosystem service of fisheries.

Contrary to previous understanding, recent observation-based studies of size spectra^27,28^, spanning from plankton to fish, have yielded unexpectedly high *TE* estimates of ~20% in the oligotrophic ocean, compared to high-nutrient waters. Although *TE* is typically expected to be ~10%, observation-based estimates range from ~5% to 15%^20^ and as wide as ~13% to 50%^21^, with higher estimates for zooplankton than for fish. Higher than expected *TE* in oligotrophic waters would explain much greater recent estimates of mesopelagic fish biomass^28^ as well as the unexpectedly high fish recruitment and meso-zooplankton production observed in the oligotrophic waters of the southern Kuroshio current^29^. Proposed mechanisms underlying this higher than expected trophic transfer have included differential enhancement of grazing compared to primary production with increasing temperature, enhanced prey capture by visual predators in clear oligotrophic waters^28^, and higher than expected primary production driven by episodic mixing^29^. Here we propose a novel mechanism, namely that heretofore under-appreciated levels of micro-scale variability, by increasing predator-prey overlap, likely substantially enhance *TE* in the oligotrophic ocean.

Many authors have reported that heterogeneity enhances species richness^2,3,6,30,31^ with an overall positive effect across taxa and regions. Most such studies found that heterogeneity consistently enhances biodiversity^32–34^, although this depends on the biodiversity metric (e.g., richness, evenness) and scale (e.g., landscape, or temporal-variations) considered. As found previously for a single NPZ model configuration^14^, our extensive simulations herein revealed that increasing micro-scale variability consistently widened the stability domain for all model configurations considered. This suggests strongly that micro-scale variability enhances potential biodiversity, by making a wider range of trait (parameter) values viable, thereby increasing the probability that different species will be able to coexist by exploiting different niches.

Gradients of micro-scale variability, such as observed for *CV*, may be important indicators of overall plankton biodiversity and the efficiency with which primary production can be transferred up the food chain. Observations from Tokyo bay revealed low values of *CV* < 1 in its interior where freshwater inputs are greatest, with an increasing gradient towards *CV* > 1 for more saline seawater. The meta-analysis of^15^ similarly found the greatest degree of observed predator-prey overlap in tropical regions, although they considered variability at larger scales. Our results presented herein were obtained using only 0-D models, which cannot account for advection, diffusion, and spatial gradients. Preliminary results (Kyohei Imamura, personal communication) with a 1-D implementation of this modelling approach have yielded similar patterns for the enhancement of trophic transfer with increasing micro-scale variability. Further studies are needed to investigate the impact of micro scale variability in spatially explicit 1-D and 3-D models.

Micro-scale variability is probably most important for sustaining plankton biodiversity in low nutrient, high salinity waters such as subtropical gyres and the stratified near-surface in tropical regions. Based on our observations and model results we hypothesise a novel answer to the “paradox of the plankton” for such calm, low-nutrient oceanic environments: Heretofore under-appreciated high levels of micro-scale variability may explain the great diversity of plankton present in these vast low-nutrient regions of the ocean. Furthermore, given the generally positive relationship found between species richness and productivity^35–37^, micro-scale variability, by enhancing biodiversity, may also indirectly contribute to sustaining primary productivity in such oligotrophic regions.

## Conclusions

Our finding, that *mm*-scale variability as quantified by the coefficient of variation for phytoplankton fluorescence (*CV*) consistently enhances trophic transfer to the highest trophic level in all model configurations considered, provides a new explanation for independent observation-based estimates of high *TE* in the oligotrophic ocean. Our modelling results also add support to previous hypotheses that environmental heterogeneity in general enhances species richness. Based on the results presented herein and previously, we conclude that:

1. Higher values of *CV* support larger stability domains for the closure models.
2. High values of *CV* enhance *TE*.

By combining observations of *CV* with our closure modeling approach, we were also able to test typically applied grazing response functions, which are a major source of structural uncertainty in ecosystem models. Of the functions tested, only the  Holling Type-II equation allowed stable model solutions over the full observed range of *CV*, suggesting that this grazing function should be preferred over other widely used alternatives. Finally, we hypothesize that *CV*, which scales directly with overall micro-scale variability, constitutes an observable index for both trophic transfer efficiency and the potential of aquatic environments to sustain plankton biodiversity, i.e. that a high value of *CV* may indicate both high *TE* and high plankton biodiversity. Further concomitant observations of micro-scale variability, environmental conditions, and plankton size spectra will be needed to validate these hypotheses and to examine more completely the effects on biodiversity in terms of evenness and trait distributions [e.g.^25^] in addition to species richness.

## Supplementary information

Appendix

## Data Availability

Observed oceanic data and the model results are available from Mamoru Tanaka (tanaka.mamoru0@gmail.com) and Anupam Priyadarshi (anupam240@gmail.com) respectively on reasonable request.
