# Supplementary material for: Micro-scale patchiness enhances trophic transfer efficiency and potential plankton biodiversity
Source: Sci Rep. 2019 Nov 21;9:17243. doi: 10.1038/s41598-019-53592-6 (PMC6872819; doi:10.1038/s41598-019-53592-6)
Supplement: Supplementary file 1 — Appendix [file 41598_2019_53592_MOESM1_ESM.pdf]

Micro-scale patchiness enhances trophic transfer efficiency and potential plankton biodiversity

Anupam Priyadarshi[a], S. Lan Smith[b], Sandip Mandal[c,d], Mamoru Tanaka[c], HidekatsuYamazaki[c*]

* Corresponding author. hide@kaiyodai.ac.jp

[a]Department of Mathematics, Institute of Science, Banaras Hindu University, Varanasi, 221005 India

[b]Earth SURFACE System Research Center, Research Institute for Global Change (RIGC), Japan Agency for Marine-Earth Science and Technology, 3173-25 Showa-machi, Kanazawa-ku, Yokohama 236-0001, Japan

[c]Department of Ocean Sciences, Tokyo University of Marine Science and Technology, Minato-ku, Tokyo 108-8477, Japan

[d]Translational Global Health and Policy Research Cell, Indian Council of Medical Research, New Delhi, 110001 India

1. Observations

Materials and methods

As described in the main text, we used the Turbulence Ocean Microstructure Acquisition Profiler-Laser (TurboMAP-L; [1]) to observe the *CV* of fluorescence. TurboMAP-L is a 2 m long and 0.12 m diameter cylinder-shaped free-fall profiler with 30 kg weight in the air. Each sensor measures undisturbed environments as TurboMAP-L falls freely downward into the ocean. Profiling speed was about 0.5 m s^{-1} in this study. The laser fluorescence sensor, whose sampling volume is 32 μL, captures *in situ* variability of the fluorescence field with an effective resolution of 2 mm [1]. We conducted a field campaign on May 24 to 25th, 2015 in Tokyo Bay, Japan (Fig. 1, and Table 1 in the main text), which is a semi-enclosed bay connected with the Pacific Ocean by the Sagami-nada Sea. The outer part of the bay is affected by the Kuroshio Current (the

western North Pacific boundary current) and the inner part of the bay is affected by freshwater inflow of about 13 billion tons annually throughout the bay [2]. This typically induces horizontal gradients of temperature and salinity, as well as of chemical (e.g. nutrients) and biological (e.g. phytoplankton abundance and community structure) characteristics [3, 4]. The horizontal gradients were observed for chlorophyll a, salinity, and temperature along the transect (Fig. S1) in the upper layer (above 30 m depth). Chlorophyll *a* concentrations were >10 $\mu g \mathrm{L}^{-1}$ for the upper 10 m layer at Stns. 5 to 7 and reached 20 µg L^{-1} at 5 m depth at Stn. 7 (Fig. S1a). On the other hand, chlorophyll *a* values were <3 $\mu g \mathrm{L}^{-1}$ at Stns. 1 to 3 (Fig. S1a). Stn. 4 had intermediate values ≈ 5 µg L^{-1}. Lower salinity and higher temperature values were observed in the inner bay: salinity and temperature at Stn. 1 were ≈ 34 PSU and ≈ 18 ℃ respectively, while these were ≈ 30 PSU and ≈ 20 ℃at Stn. 7 (Fig. S1b,c). For deep layers (<30 m depth; only at Stn. 1 and 2), chlorophyll *a* concentrations were <1 $\mu g \mathrm{L}^{-1}$ on average, salinity was almost homogeneous ≈ 34.5 PSU, and temperature was ≈ 16 ℃ at 30 m depth and decreased with depth at a rate of about −0.04 ℃ m^{-1} to 100m depth. By water mass analysis, we found that Stns. 1 to 3 had similar water mass properties (relatively saline and cold), and the water became less saline and warmer going towards Stns. 4 through Stn. 7 (Fig. S2).

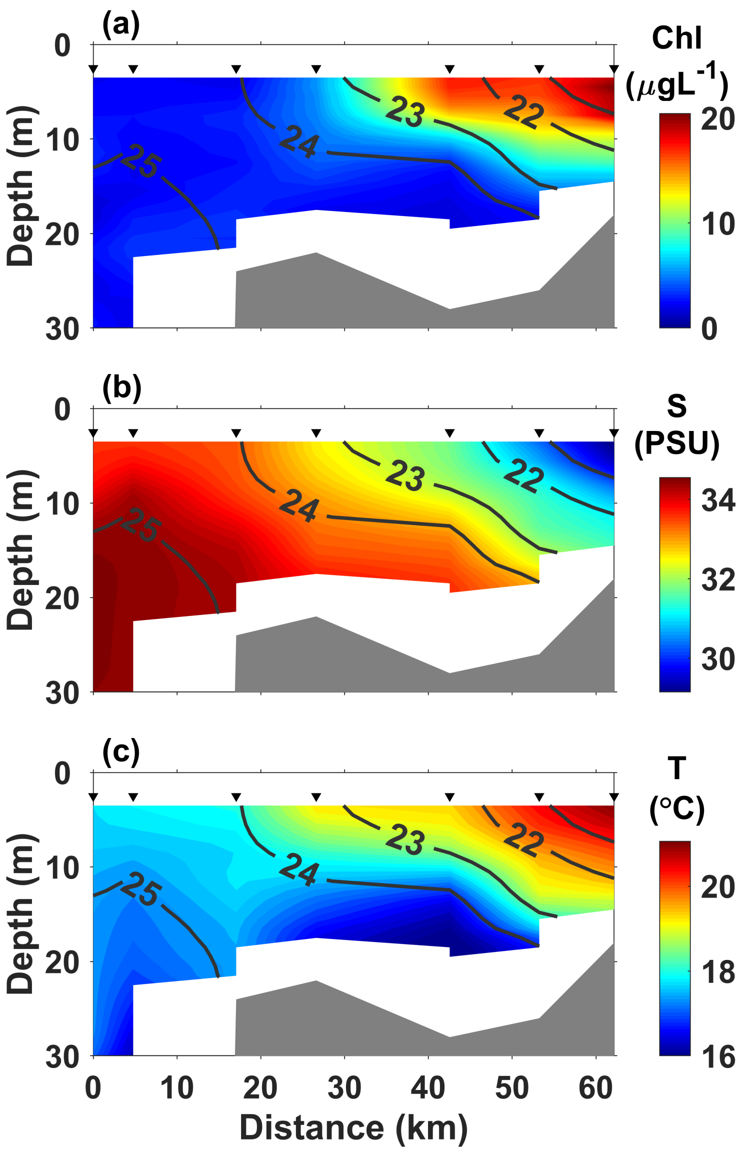

Fig. S1. Vertical cross sections for (a) chlorophyll *a*, (b) salinity, and (c) temperature along the transect (Fig. 1a in the main text). Filled black triangles indicate locations of Stn. 1 to Stn. 7 (left to right), with contours (thick black lines) of potential density. The x-axes show distance from Stn. 1. The bottom topography is shown by the filled grey area.

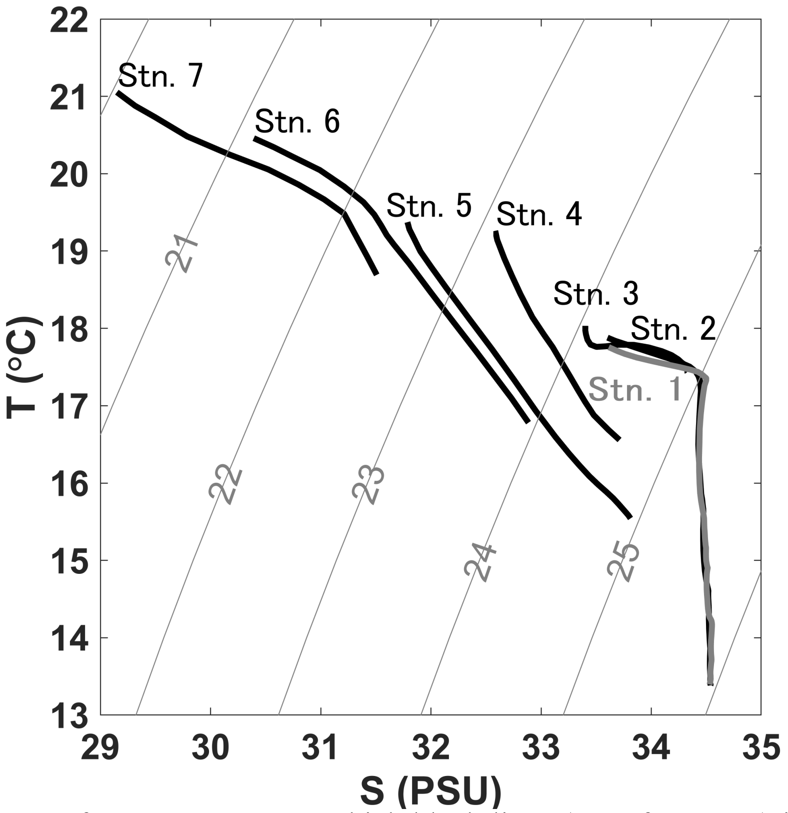

Fig. S2. T-S diagram for *Stn. 1 to Stn. 7.* Thick black lines (grey for Stn. 1) indicate the profiles shown in the Fig. S1, with contours for potential density (thin grey lines).

2. Mathematical models

Closure models

The ecological interactions of nutrient (*N*), phytoplankton (*P*), zooplankton (Z) and detritus (D) in the generalized form are discussed in main text equations (3-6) which were developed based on the mean-field approach (considering only first central moment, ignoring the higher central moments effects [5-7]).

Closure models are derived using the Reynolds decomposition as is widely used in turbulence studies. Variables as functions of time (*t*) and space (*s*) are each decomposed into mean and fluctuating components as,

$$N(s,t) = N_0(s,t) + N'(s,t), \quad P(s,t) = P_0(s,t) + P'(s,t)$$

$$Z(s,t) = Z_0(s,t) + Z'(s,t), \quad D(s,t) = D_0(s,t) + D'(s,t) \tag{S1}$$

Here, the fluctuating components may be either positive or negative. Observed phytoplankton profiles reveal that most of these fluctuating components are small relative to the mean value, with few components having values greater than the mean value. According to field measurements based on a laser fluorescence probe, P' follows a *Gumbel extreme value* probability distribution [1, 8, 9]. A large fraction of signals from this probability distribution appears below the mean. Therefore, we use the Taylor expansion around the mean of each variable and retain the terms up to second order only to approximate the temporal variation of variance and covariance in the closure model. Observations of microscale profiles of phytoplankton using different instruments (such as Seapoint fluorometer, Light Emitting Diode (LED) sensor, Laser sensor), suggest that at a particular time, the depth-average of the fluctuating components is zero [10, 11]. Therefore, ($\langle N'(s)\rangle = \langle P'(s)\rangle = \langle Z'(s)\rangle = \langle D'(s)\rangle = 0$), while the temporal average can be nonzero, which also implies $\langle N(s)\rangle = N_0(s)$, $\langle P(s)\rangle = P_0(s)$, $\langle Z(s)\rangle = Z_0(s)$ and $\langle D(s)\rangle = D_0(s)$. With these assumptions, we

can derive equations for the temporal variation of mean and fluctuating components for all variables.

Generalized closure model framework

In order to develop the generalized closure model, assume the nutrient uptake term as $f = F_N(N)P$ and consider the following functions,

$$
\begin{aligned}
&f_1 = f(N_0, P_0) = \frac{N_0 P_0}{K + N_0}, \quad f_2 = \frac{\partial f}{\partial N} = \frac{K P_0}{(K + N_0)^2}, \quad f_3 = \frac{\partial f}{\partial P} = \frac{N_0}{(K + N_0)}, \\
&f_4 = \frac{1}{2}\frac{\partial^2 f}{\partial N^2} = \frac{-K P_0}{(K + N_0)^3}, \quad f_5 = \frac{\partial^2 f}{\partial N \, \partial P} = \frac{K}{(K + N_0)^2}, \quad f_6 = \frac{1}{2}\frac{\partial^2 f}{\partial P^2} = 0
\end{aligned} \qquad \text{(S2)}
$$

Similarly, for the Z-grazing response function, define $g_p = G_P(R, \hat{\mu}, P)\, Z$ and consider the following functions ,

$$
g_1 = g_P(P_0, Z_0), \quad g_2 = \frac{\partial g_P}{\partial P}, \quad g_3 = \frac{\partial g_P}{\partial Z}, \quad g_4 = \frac{1}{2}\frac{\partial^2 g_P}{\partial P^2}, \quad g_5 = \frac{\partial^2 g_P}{\partial P \partial Z}, \quad g_6 = \frac{1}{2}\frac{\partial^2 g_P}{\partial Z^2} \qquad \text{(S3)}
$$

The generalized set of equations is then:

Mean Equations:

$$
\frac{dN_0}{dt} = -\nu_{\max}(f_1 + f_4 \langle N'^2 \rangle + f_5 \langle N'P' \rangle) + M_{ZN}\, Z_0 + \gamma_M D_0 \qquad \text{(S4a)}
$$

$$
\frac{dP_0}{dt} = \nu_{\max}(f_1 + f_4 \langle N'^2 \rangle + f_5 \langle N'P' \rangle) - M_P\, P_0 - (g_1 + g_4 \langle P'^2 \rangle + g_5 \langle P'Z' \rangle) \qquad \text{(S4b)}
$$

$$
\frac{dZ_0}{dt} = (1-\gamma)\,(g_1 + g_4 \langle P'^2 \rangle + g_5 \langle P'Z' \rangle) - M_{ZN}\, Z_0 - M_{ZD}\, Z_0 \qquad \text{(S4c)}
$$

$$
\frac{dD_0}{dt} = M_P\, P_0 + \gamma\,(g_1 + g_4 \langle P'^2 \rangle + g_5 \langle P'Z' \rangle) + M_{ZD}\, Z_0 - \gamma_M D_0 \qquad \text{(S4d)}
$$

Variance Equations:

$$
\frac{d\langle N'^2 \rangle}{dt} = -2\nu_{\max}(f_2 \langle N'^2 \rangle + f_3 \langle N'P' \rangle) + 2\, M_{ZN}\, \langle N'Z' \rangle + 2\, \gamma_M\, \langle N'D' \rangle \qquad \text{(S5a)}
$$

$$\frac{d\langle P'^2\rangle}{dt} = 2v_{\max}(f_2\langle N'P'\rangle + f_3\langle P'^2\rangle) - 2M_P\langle P'^2\rangle - 2(g_2\langle P'^2\rangle + g_3\langle P'Z'\rangle) \tag{S5b}$$

$$\frac{d\langle Z'^2\rangle}{dt} = 2(1-\gamma)(g_2\langle P'Z'\rangle + g_3\langle Z'^2\rangle) - 2M_{ZN}\langle Z'^2\rangle - 2M_{ZD}\langle Z'^2\rangle \tag{S5c}$$

$$\frac{d\langle D'^2\rangle}{dt} = 2M_P\langle P'D'\rangle + 2\gamma(g_2\langle P'D'\rangle + g_3\langle Z'D'\rangle) + 2M_{ZD}\langle Z'D'\rangle - 2\gamma_M\langle D'^2\rangle \tag{S5d}$$

Covariance Equations:

$$\begin{aligned}\frac{d<N'P'>}{dt} &= v_{\max}(f_3(<N'P'> - <P'^2>) + f_2(<N'^2> - <N'P'>)) - M_P <N'P'> + M_{ZN} <P'Z'> \\ &+ \gamma_M <P'D'> - (g_2 <N'P'> + g_3 <N'Z'>)\end{aligned} \tag{S6a}$$

$$\begin{aligned}\frac{d<N'Z'>}{dt} &= -v_{\max}(f_3 <P'Z'> + f_2 <N'Z'>) + M_{ZN}(<Z'^2> - <N'Z'>) \\ &- M_{ZD} <N'Z'> + \gamma_M <D'Z'> + (1-\gamma)(g_2 <N'P'> + g_3 <N'Z'>)\end{aligned} \tag{S6b}$$

$$\begin{aligned}\frac{d<N'D'>}{dt} &= -v_{\max}(f_3 <P'D'> + f_2 <N'D'>) + M_P <N'P'> + M_{ZN} <Z'D'> + M_{ZD} <N'Z'> \\ &+ \gamma_M[<D'^2> - <N'D'>] + \gamma(g_2 <N'P'> + g_3 <N'Z'>)\end{aligned} \tag{S6c}$$

$$\begin{aligned}\frac{d<P'Z'>}{dt} &= v_{\max}(f_3 <P'Z'> + f_2 <N'Z'>) - (M_P + M_{ZN} + M_{ZD}) <P'Z'> \\ &+ g_2((1-\gamma) <P'^2> - <P'Z'>) + g_3((1-\gamma) <P'Z'> - <Z'^2>)\end{aligned} \tag{S6d}$$

$$\begin{aligned}\frac{d<P'D'>}{dt} &= v_{\max}(f_3 <P'D'> + f_2 <N'D'>) + M_P(<P'^2> - <P'D'>) + M_{ZD} <P'Z'> \\ &- \gamma_M <P'D'> + g_2(\gamma <P'^2> - <P'D'>) + g_3(\gamma <P'Z'> - <Z'D'>)\end{aligned} \tag{S6e}$$

$$\begin{aligned}\frac{d<Z'D'>}{dt} &= M_P <P'Z'> + M_{ZD}(<Z'^2> - <Z'D'>) - (M_{ZN} + \gamma_M) <Z'D'> \\ &+ g_2(\gamma <P'Z'> + (1-\gamma) <P'D'>) + g_3(\gamma <Z'^2> + (1-\gamma) <Z'D'>)\end{aligned} \tag{S6f}$$

Dimensionless form of closure model

In this section, we present the dimensionless form of the generalized mean-field equations, generalized variance and co-variances equations for plankton models. The dimensionless form is quite useful for mathematical analysis as well as for numerical simulations.

The following conservation relationships are obtained from the closure model equations (S4a-S6f):

$$N_0 + P_0 + Z_0 + D_0 = \text{Constant} = A \qquad \text{(S7)}$$

$$\begin{aligned}&\langle P'^2\rangle + \langle Z'^2\rangle + \langle N'^2\rangle + \langle D'^2\rangle + 2(\langle N'P'\rangle + \langle N'Z'\rangle + \\ &\langle N'D'\rangle + \langle P'Z'\rangle + \langle P'D'\rangle + \langle Z'D'\rangle) = Cons\tan t = B\end{aligned} \qquad \text{(S8)}$$

A dimensionless quantity called **"micro-scale variability, β"** is defined as the ratio of total fluctuating strength (i.e., the sum of variances and covariances), B, to the square of the total mean-field nutrient concentration, A:

$$\beta = \frac{B}{A^2} \qquad \text{(S9)}$$

We used the following scaled variables and the following dimensionless quantities to obtain generalized dimensionless form of plankton models:

$$n_0 = \frac{N_0}{A}, p_0 = \frac{P_0}{A}, z_0 = \frac{Z_0}{A}, d_0 = \frac{D_0}{A} \text{ and } t' \rightarrow v_{\max} t \qquad \text{(S10a)}$$

$$\langle n'^2\rangle = \left\langle \frac{N'^2}{B} \right\rangle, \langle p'^2\rangle = \left\langle \frac{P'^2}{B} \right\rangle, \langle z'^2\rangle = \left\langle \frac{Z'^2}{B} \right\rangle, \langle d'^2\rangle = \left\langle \frac{D'^2}{B} \right\rangle \qquad \text{(S10b)}$$

$$\langle n'p'\rangle = \left\langle \frac{N'P'}{B} \right\rangle, \langle n'z'\rangle = \left\langle \frac{N'Z'}{B} \right\rangle, \langle n'd'\rangle = \left\langle \frac{N'D'}{B} \right\rangle \qquad \text{(S10c)}$$

$$\langle p'z'\rangle = \frac{\langle P'Z'\rangle}{B}, \langle p'd'\rangle = \frac{\langle P'D'\rangle}{B}, \langle z'd'\rangle = \frac{\langle Z'D'\rangle}{B} \qquad \text{(S10d)}$$

$$m_{zn} = \frac{M_{ZV}}{V_{\max}}, m_{zd} = \frac{M_{ZD}}{V_{\max}}, m_p = \frac{M_P}{V_{\max}}, r = \frac{R}{V_{\max}}, k = \frac{K}{A}, \gamma_m = \frac{\gamma_M}{V_{\max}}, \mu = \frac{\hat{\mu}}{A} \qquad \text{(S10e)}$$

The following dimensionless conservation equations can be obtained:

$$n_0 + p_0 + z_0 + d_0 = 1 \qquad \text{(S10f)}$$

$$\langle n'^2\rangle+\langle p'^2\rangle+\langle z'^2\rangle+\langle d'^2\rangle+2(\langle n'p'\rangle+\langle n'z'\rangle+\langle n'd'\rangle+\langle p'd'\rangle+\langle z'd'\rangle)=1 \quad \text{(S10g)}$$

Accordingly, the nutrient uptake term $f_n=\frac{n}{k+n}$ and three different Z-grazing functional responses are:

(i) $g_p(r,\mu,p)=r\,p$

(ii) $g_p(r,\mu,p)=\frac{r\,p}{\mu+p}$

(iii) $g_p(r,\mu,p)=\frac{rp^2}{\mu^2+p^2}$

The dimensionless form of these functions f_i and g_i which are defined in (S2) and (S3) above, are:

$$f_1=\frac{n_0p_0}{k+n_0}, f_2=\frac{k\,p_0}{(k+n_0)^2}, f_3=\frac{n_0}{k+n_0}, f_4=\frac{-k\,p_0}{(k+n_0)^3}, f_5=\frac{k}{(k+n_0)^2}, f_6=0 \quad \text{(S11)}$$

The following expressions are used to represent each of the Z-grazing rate expressions, respectively:

(i) **Linear Z-grazing**

$$g_1=r\,p_0\,z_0, g_2=r\,z_0, g_3=r\,p_0,\; g_4=0, g_5=r, g_6=0 \quad \text{(S12a)}$$

(ii) **Holling Type II Z-grazing**

$$g_1=\frac{r\,p_0\,z_0}{\mu+p_0}, g_2=\frac{r\,\mu\,z_0}{(\mu+p_0)^2}, g_3=\frac{r\,p_0}{\mu+p_0},\; g_4=\frac{-r\,\mu\,z_0}{(\mu+p_0)^3}, g_5=\frac{r\,\mu}{(\mu+p_0)^2}, g_6=0 \quad \text{(S12b)}$$

(ii) **Holling Type III Z-grazing**

$$g_1=\frac{r\,p_0^2\,z_0}{\mu^2+p_0^2}, g_2=\frac{2\,r\,\mu^2\,p_0z_0}{(\mu^2+p_0^2)^2}, g_3=\frac{r\,p_0^2}{\mu^2+p_0^2},$$
$$g_4=\frac{r\,\mu^2(\mu^2-3p_0^2)\,z_0}{(\mu^2+p_0^2)^3},\quad g_5=\frac{2\,r\,\mu^2p_0}{(\mu^2+p_0^2)^2}, g_6=0 \quad \text{(S12c)}$$

Generalized mean-field equations

$$\frac{d\,p_0}{dt} = f_1 + f_5\,\beta\langle n'p'\rangle + f_4\,\beta\langle n'^2\rangle - m_p\,p_0 - (g_1 + g_5\,\beta\,\langle p'z'\rangle + g_4\,\beta\langle p'^2\rangle) \tag{S13a}$$

$$\frac{d\,n_0}{dt} = -(f_1 + f_5\,\beta\langle n'p'\rangle + f_4\,\beta\langle n'^2\rangle) + m_{zn}\,z_0 + \gamma_m d_0 \tag{S13b}$$

$$\frac{d\,z_0}{dt} = (1-\gamma)\,(g_1 + g_5\,\beta\,\langle p'z'\rangle + g_4\,\beta\langle p'^2\rangle) - m_{zn}\,z_0 - m_{zd}\,z_0 \tag{S13c}$$

$$\frac{d(d_0)}{dt} = \gamma\,(g_1 + g_5\,\beta\,\langle p'z'\rangle + g_4\,\beta\langle p'^2\rangle) + m_p\,p_0 + m_{zd}\,z_0 - \gamma_m d_0 \tag{S13d}$$

Generalized variance equations

$$\frac{d\langle n'^2\rangle}{dt} = 2[-f_3\langle n'p'\rangle - f_2\,\langle n'^2\rangle + m_{zn}\,\langle n'z'\rangle + \gamma_m\,\langle n'd'\rangle] \tag{S14a}$$

$$\frac{d\langle p'^2\rangle}{dt} = 2[f_3\langle p'^2\rangle + f_2\,\langle n'p'\rangle - m_p\,\langle p'^2\rangle - (g_2\,\langle p'^2\rangle + g_3\,\langle p'z'\rangle)] \tag{S14b}$$

$$\frac{d\langle z'^2\rangle}{dt} = 2[(1-\gamma)(g_2\langle p'z'\rangle + g_3\,\langle z'^2\rangle) - m_{zn}\,\langle z'^2\rangle - m_{zd}\,\langle z'^2\rangle] \tag{S14c}$$

$$\frac{d\langle d'^2\rangle}{dt} = 2[\gamma\,(g_2\,\langle p'd'\rangle + g_3\,\langle z'd'\rangle) + m_p\,\langle p'd'\rangle + m_{zd}\,\langle z'd'\rangle - \gamma_m\,\langle d'^2\rangle] \tag{S14d}$$

Generalized covariance equations

$$\begin{aligned}\frac{d\langle n'p'\rangle}{dt} &= f_3(\langle n'p'\rangle - \langle p'^2\rangle) + f_2\,(\langle n'^2\rangle - \langle n'p'\rangle) - m_p\,\langle n'p'\rangle + m_{zn}\,\langle p'z'\rangle \\ &\quad + \gamma_m\,\langle p'd'\rangle - (g_2\langle n'p'\rangle + g_2\langle n'z'\rangle)\end{aligned} \tag{S15a}$$

$$\begin{aligned}\frac{d\langle n'z'\rangle}{dt} &= -f_3\langle p'z'\rangle - f_2\,\langle n'z'\rangle - m_{zn}(\,\langle n'z'\rangle - \langle z'^2\rangle) + \gamma_m\,\langle z'd'\rangle \\ &\quad - m_{zd}\,\langle n'z'\rangle + (1-\gamma)\,\,(g_2\langle n'p'\rangle + g_3\langle n'z'\rangle)\end{aligned} \tag{S15b}$$

$$\begin{aligned}\frac{d\,\langle p'z'\rangle}{dt} &= f_3\,\langle p'z'\rangle + f_2\,\langle n'z'\rangle - (m_p + m_{zn} + m_{zd})\,\langle p'z'\rangle \\ &\quad + \,g_2[(1-\gamma)\,\langle p'^2\rangle - \langle p'z'\rangle] + g_3[(1-\gamma)\,\langle p'z'\rangle - \langle z'^2\rangle]\end{aligned} \tag{S15c}$$

$$\frac{d\langle p'd'\rangle}{dt} = f_3\langle p'd'\rangle + f_2\langle n'd'\rangle + m_p(\langle p'^2\rangle - \langle p'd'\rangle) + m_{zd}\langle p'z'\rangle - \gamma_m\langle p'd'\rangle + g_2[\gamma\langle p'^2\rangle - \langle p'd'\rangle] + g_3[\gamma\langle p'z'\rangle - \langle z'd'\rangle] \quad \text{(S15d)}$$

$$\frac{d\langle z'd'\rangle}{dt} = g_2[\gamma\langle p'z'\rangle + (1-\gamma)\langle p'd'\rangle] + g_3[\gamma\langle z'^2\rangle + (1-\gamma)\langle z'd'\rangle] + m_p\langle p'z'\rangle + m_{zd}(\langle z'^2\rangle - \langle z'd'\rangle) - m_{zn}\langle z'd'\rangle - \gamma_m\langle z'd'\rangle \quad \text{(S15e)}$$

$$\frac{d\langle n'd'\rangle}{dt} = -f_3\langle p'd'\rangle - f_2\langle n'd'\rangle + m_p\langle n'p'\rangle - \gamma_m(\langle n'd'\rangle - d'^2\rangle) + \gamma(g_2\langle n'p'\rangle + g_3\langle n'z'\rangle) + m_{zn}\langle z'd'\rangle + m_{zd}\langle n'z'\rangle \quad \text{(S15f)}$$

Table S1. Parameter values and ranges

**Parameter**	**Description**	**Reported value**	**Dimensionless Quantity**	**Parameter range**
A	Sum of total mean nitrogen contain	2 μg N l^{-1}[12]	------	------
v_{max}	P maximum growth rate	2 day^{-1} [12, 13]	------	0.5-2.0
K	Half-saturation uptake rate	1-1.4 μg N l^{-1} [14-16]	$k = K/A$	0-5
M_{ZN}	Z-biomass loss to Nitrogen	0.2-2.0 day^{-1} [16, 21]	$m_{zn} = M_{ZN}/v_{max}$	0-1
M_{ZD}	Z-biomass loss to Detritus	0.2-2.0 day^{-1} [16, 21]	$m_{zd} = M_{ZD}/v_{max}$	0-1
M_P	Phytoplankton Mortality	0.07-0.8 day^{-1} [15-17]	$m_p = M_p/v_{max}$	0-1
R	Zooplankton grazing rate	0.6-1.4 day^{-1} [17-19]	$r = RA/v_{max}$ (for linear case [11]) $r = R/v_{max}$	0-5
γ_M	Detritus remineralisation rate	0.2-2.0 day^{-1} [16, 17, 20, 21]	$\gamma_m = \gamma_M / v_{max}$	0-0.5
γ	Z assimilation efficiency	0.3	γ	0.3

3. Numerical Simulations:

Parameter range

Suitable choices of biologically feasible parameters (Table S1) were chosen from the various empirical and theoretical plankton literatures [12-21] to carry out the numerical simulations:

$$r = 1.0, m_p = 0.4, m_{zn} = 0.15, m_{zd} = 0.07,$$
$$k = 0.5, \gamma_m = 0.5, \gamma = 0.3 \qquad \text{(Parameters)}$$

Time series analysis

The NPZD plankton model has four components, each of which may dominate under different conditions. No matter which component dominates as a fraction of total *N*, increasing micro-scale variability enhances the biomass of the highest trophic level (Fig. S3a-S3d). The same was previously observed for the NPZ model [11].

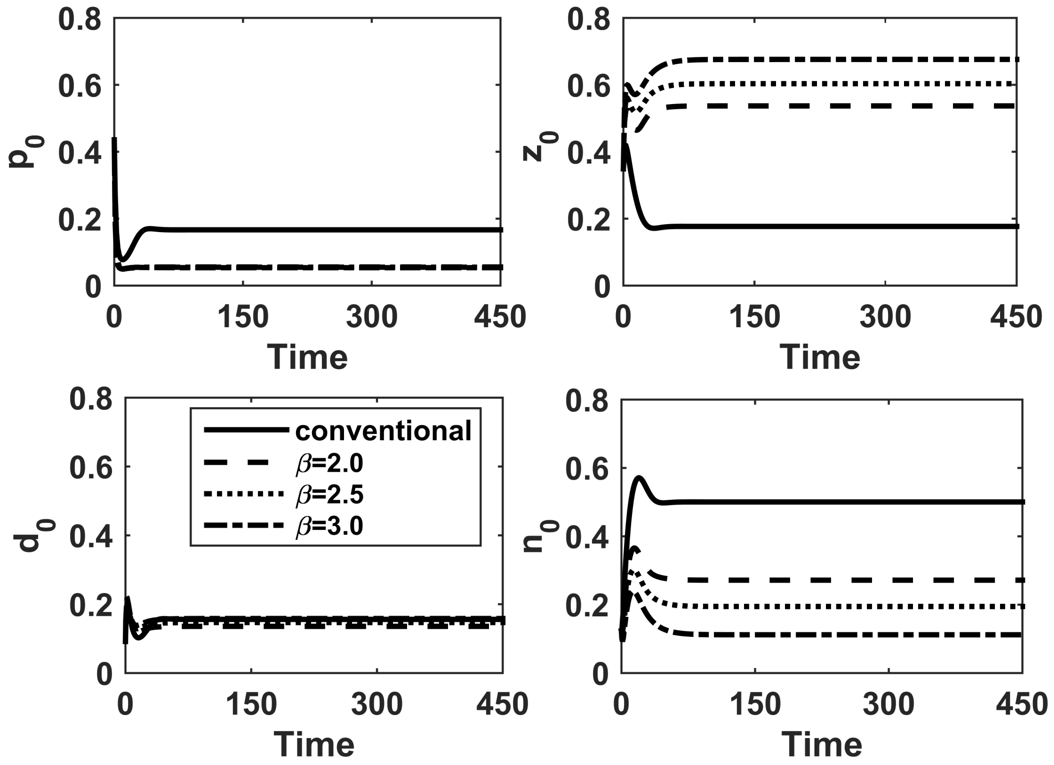

Fig. S3a. ***P*-dominating case**: The time evolution of each model component for different levels of micro-scale variability (β), under conditions such that the *P*-component dominates the fraction of total *N* in the model.

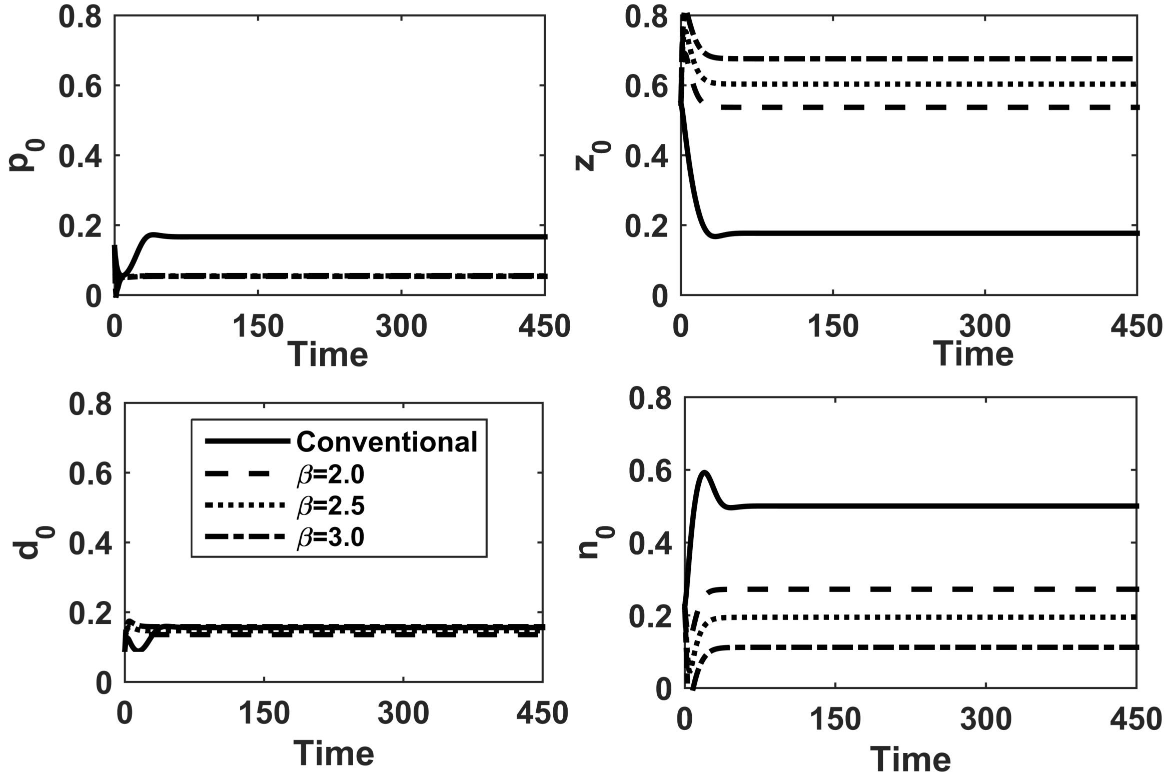

Fig. S3b. *Z*-dominating case: The time evolution of each model component for different levels of micro-scale variability (β), under conditions such that the *Z*- component dominates the fraction of total *N* in the model.

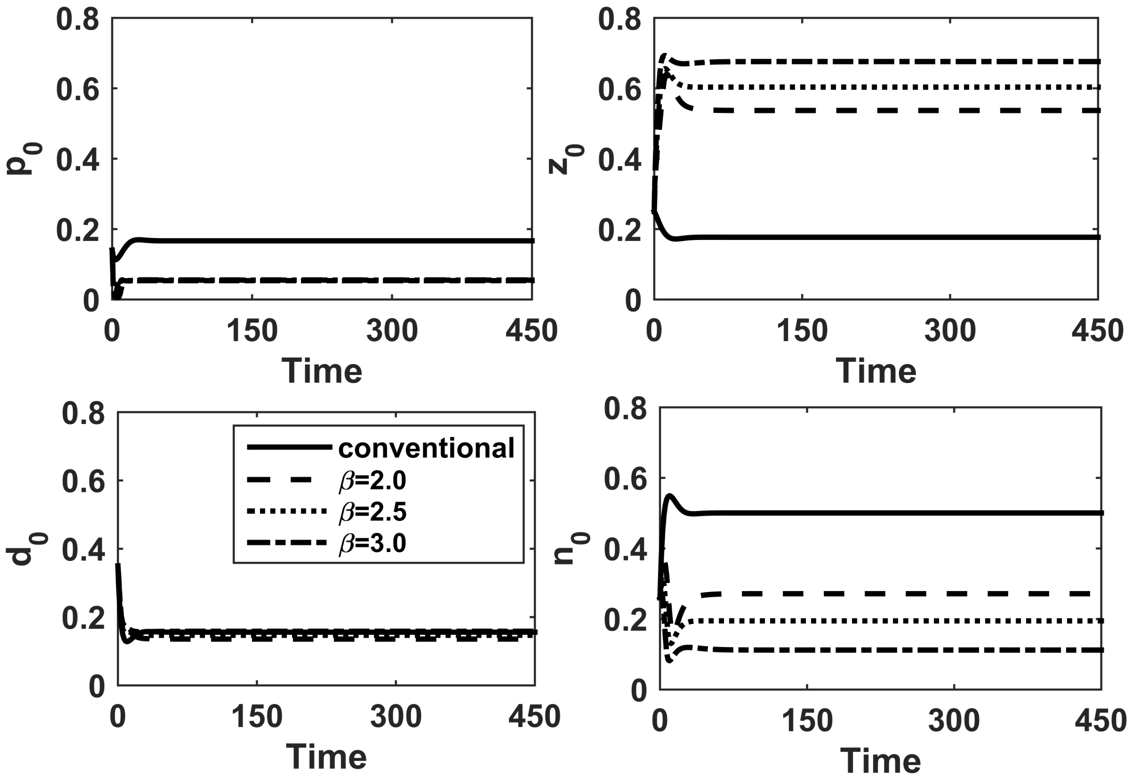

Fig. S3c. *D*-dominating case: The time evolution of each model component for different levels of micro-scale variability (β), under conditions such that the *D*- component dominates the fraction of total *N* in the model.

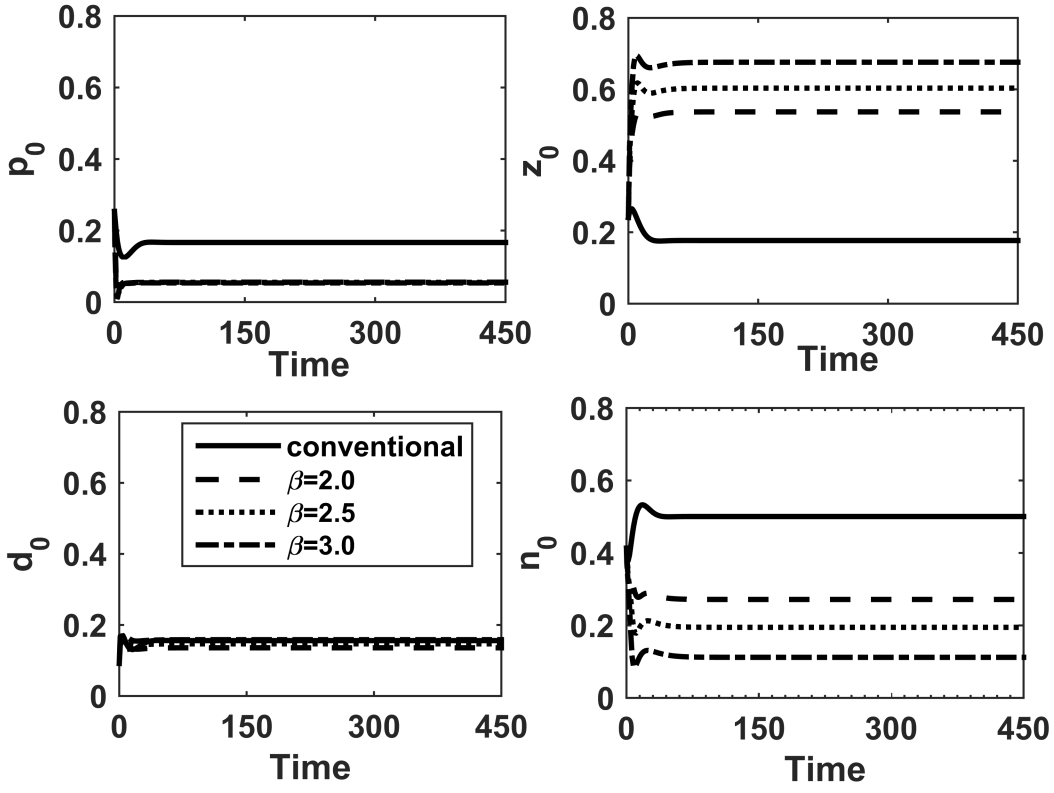

Fig. S3d. *N*-dominating case: The time evolution of each model component for different levels of micro-scale variability (β), under conditions such that the *N*- component dominates the fraction of total *N* in the model.

One-parameter bifurcation with parameters (NPZD closure model)

For one-parameter bifurcation, we use the "continuation algorithm" [22] to examine qualitative changes in the dynamics with respect to each parameter of interest. Basically, it is initiated with a suitable steady state solution $x_t(\mu_0)$ at $\mu = \mu_0$ of the system $f(x, \mu)=0$. The aim of this algorithm is to estimate the new stationary point at $\mu = \mu_0 + \Delta\mu$ starting from the earlier one. The continuation algorithm aims to trace the loci of the solutions of algebraic equations for a given first tentative value. Given that $n_0 + p_0 + z_0 + d_0 = 1$, dynamics are only depicted for *p*, *z*

and d in the figures to avoid redundancy and allow better visualization. One-parameter bifurcation diagrams showing the change in biomass with respect to each of the six parameters of parameter set (Parameters) are drawn in the Fig. S4. The biomass of phytoplankton, p, does not depend on parameters k, m_p and γ_m. However, p increases with parameters m_{zn} and m_{zd} while it decreases with increasing parameter r. Biomass of detritus increases with parameters m_p, m_{zn} and m_{zd} while it decreases with increasing parameter r, k and γ_m. Zooplankton biomass increases with parameters r and γ_m, and decreases with increasing parameters k, m_p, m_{zn} and m_{zd}.

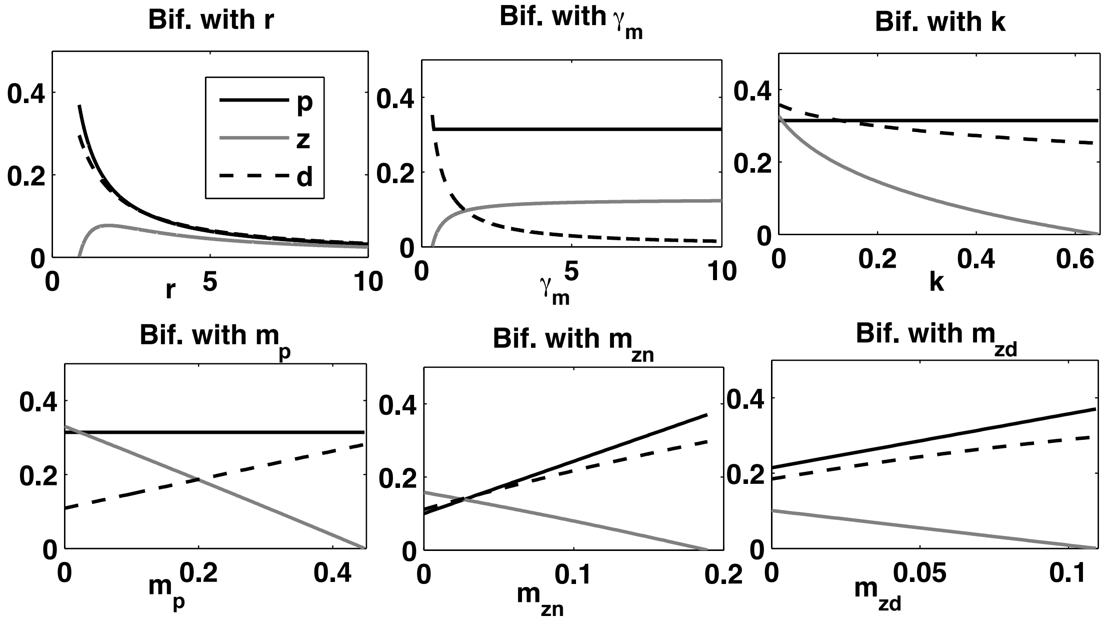

Fig. S4. Conventional NPZD model: variation in biomass of *p, z* and *d* with respect to model parameters: *Z*-grazing rate *r,* half-saturation constant *k*, *P*-mortality rate m_p, *Z*-mortality rate m_{zn}, Z-loss rate m_{zd}, and detritus remineralization efficiency γ_m.

Impact of variability on plankton dynamics

In the NPZD closure model, micro-scale variability, β, enhances zooplankton biomass, to levels greater than obtained with the conventional NPZD model (Fig. S5). The effect of micro-scale variability, β, on plankton biomass is shown in Fig. S6 for different values of Z-grazing rate (*r*). Zooplankton biomass depends more strongly on β with moderate Z-grazing rate than with higher Z-grazing rate (*r*) (Fig. S6).

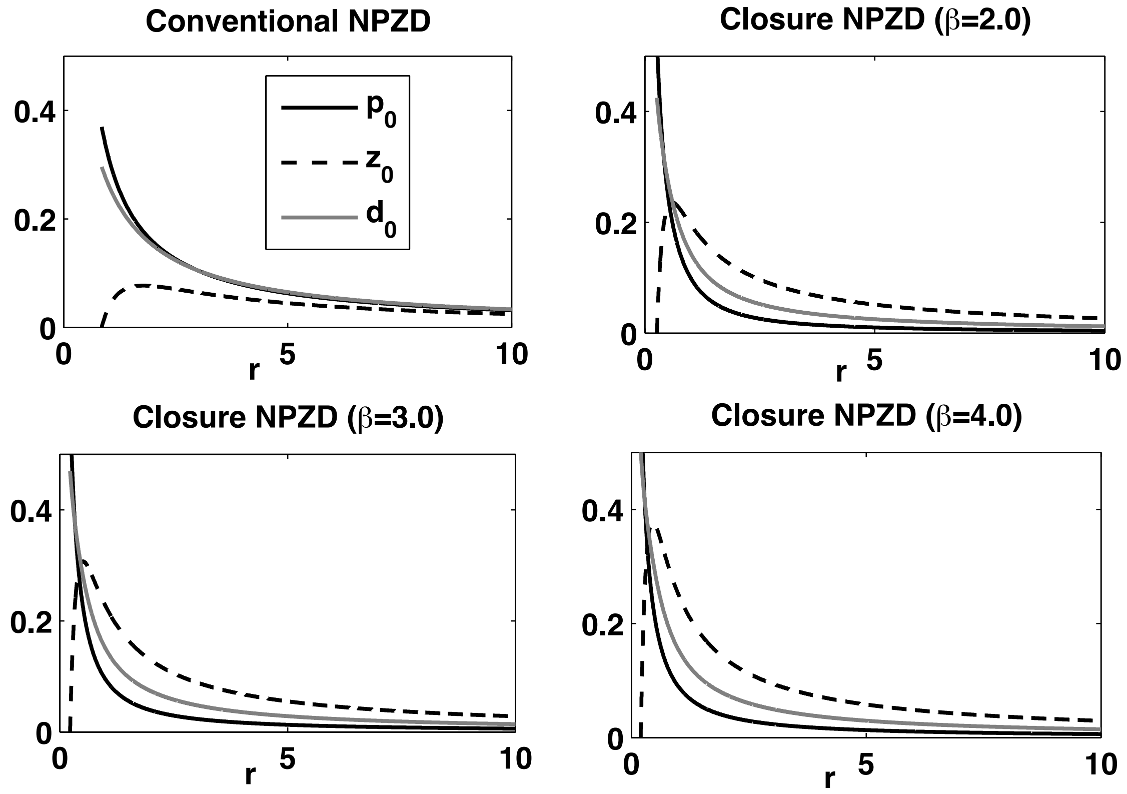

Fig. S5. The variation in dimensionless biomass of *p, z* and *d* with respect to *Z*-grazing rate *r* is depicted for the conventional NPZD model as well as for $\beta = 2.0, 3.0$ and 4.0 in the NPZD closure model. The Z-biomass is higher in the NPZD closure model than the conventional model and it increases with increasing micro-scale variability β in the NPZD closure model.

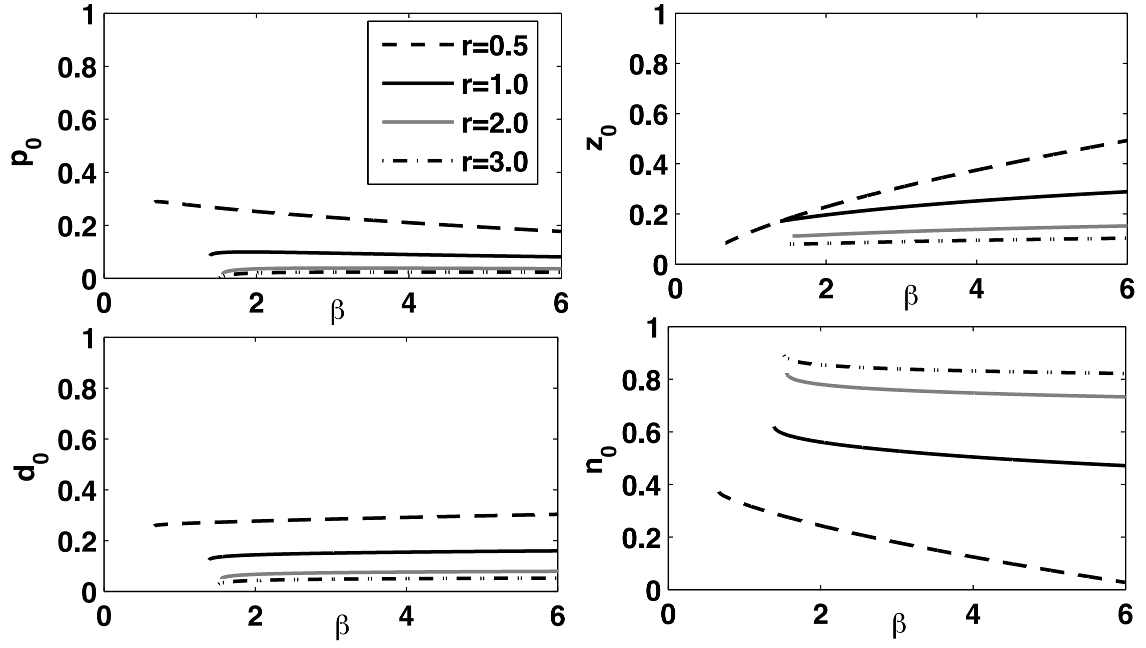

Fig. S6. The one-parameter bifurcation diagram for dimensionless mean-field values of n_o, p_o, z_o and d_o with respect to β is shown for the NPZD closure model at different *Z*-grazing rate *r*.

Parameter domain for stable solutions increases with increasing variability

The NPZD plankton dynamics and the stability of steady state solutions both depend on the parameter combinations used in the model. The stability domain (over parameter space) is shown as the shaded region in Figs. S7-S10 for different parameter combinations. Compared to the conventional NPZD model, the stability region is wider for the NPZD closure model, and it increases with the level of variability β, for all combinations of parameter values considered.

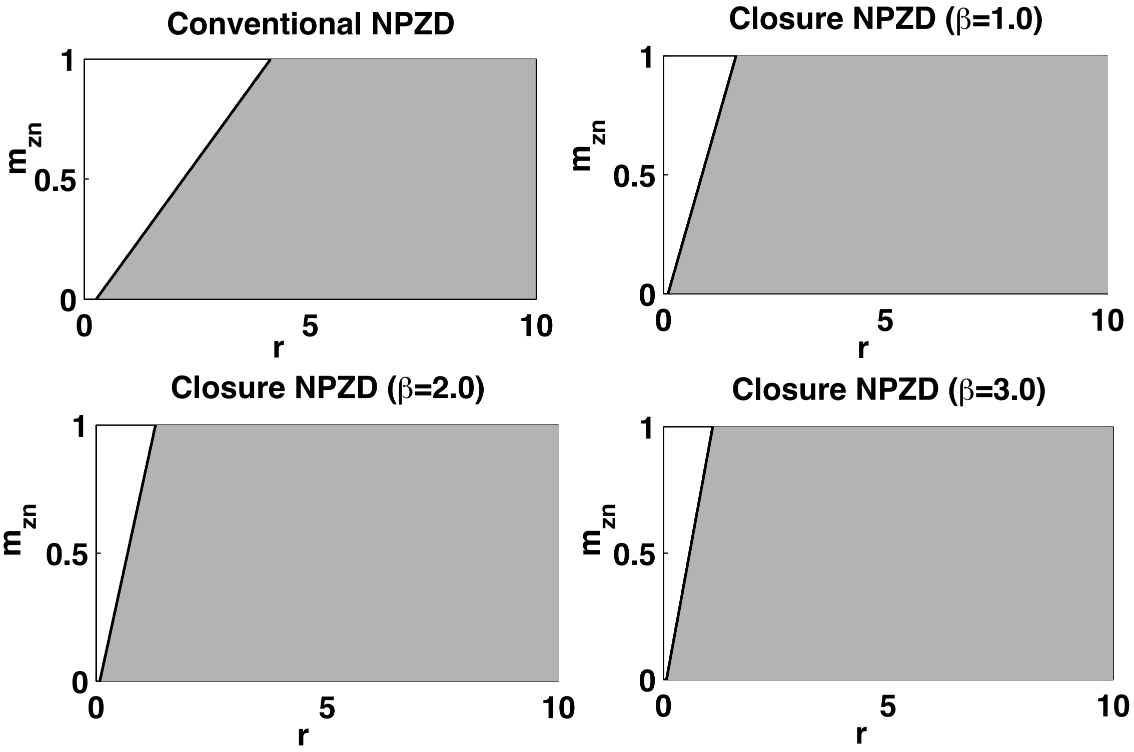

Fig. S7. The parameter region (shaded area) for stable steady states and coexistence of *N, P, Z* and *D* in the NPZD model is shown in the Two-parameter (r, m_{zn}) bifurcation diagram. The stability region increases with increasing micro-scale variability, β.

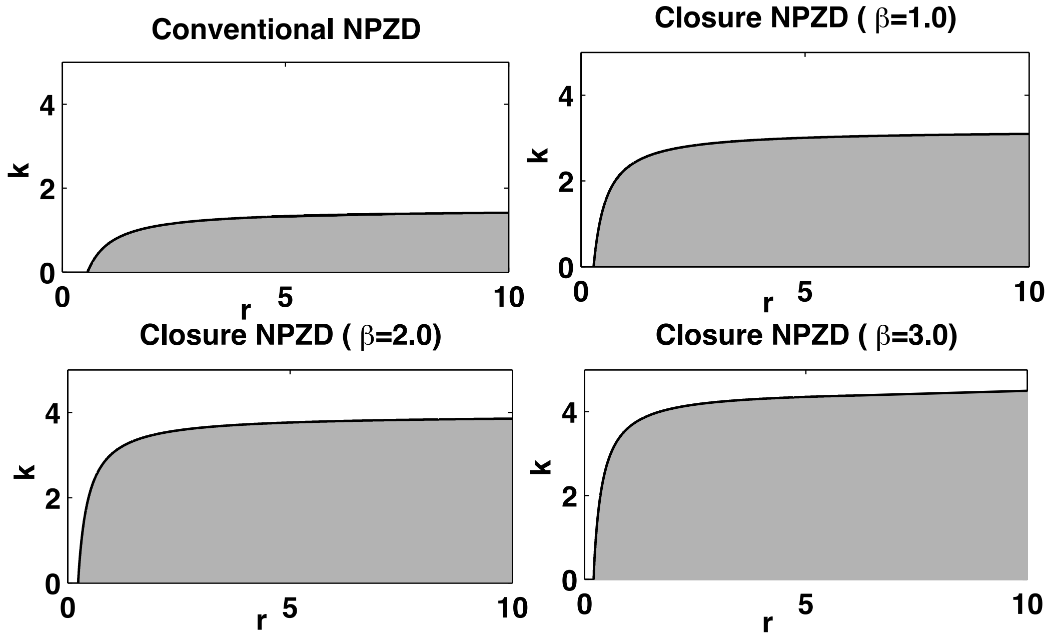

Fig. S8. The parameter region (shaded area) for stable steady states and coexistence of *N, P, Z* and *D* in the NPZD model is drawn in the Two-parameter (*r, k*) bifurcation diagram. The stability region increases with increasing micro-scale variability, β.

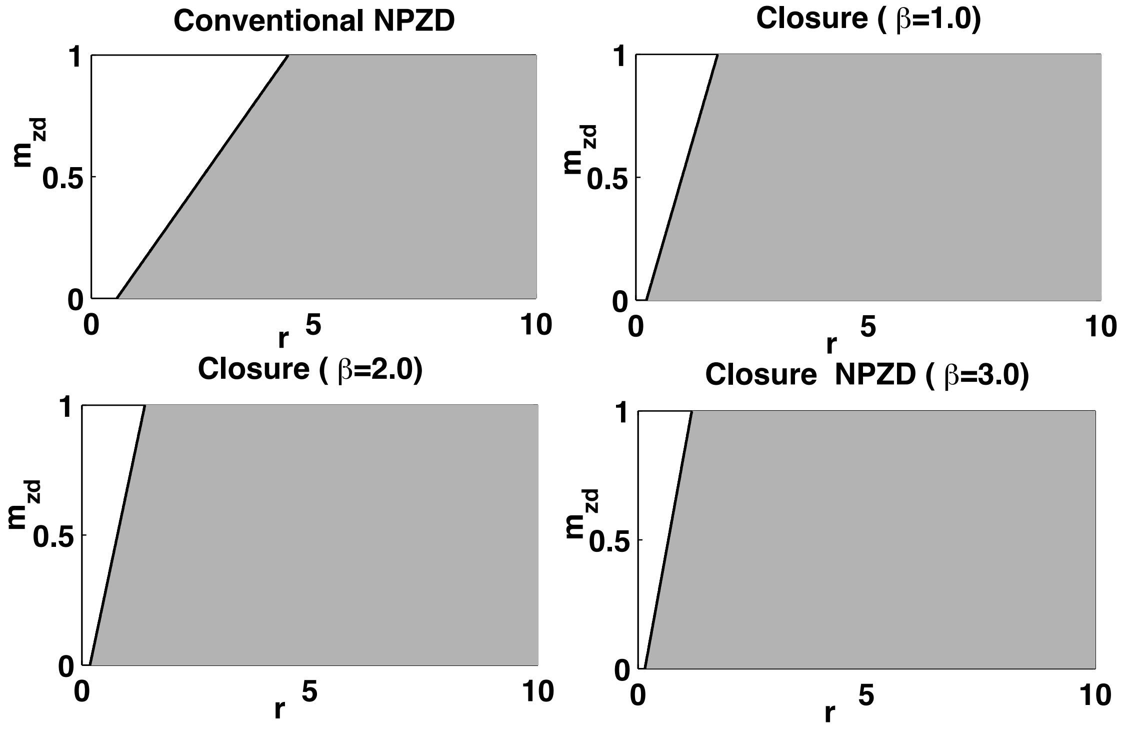

Fig. S9. The parameter region (shaded area) for stable steady states and coexistence of *N, P, Z* and *D* in the NPZD model is drawn in the Two-parameter (r, m_{zd}) bifurcation diagram. The stability region increases with increasing micro-scale variability, β.

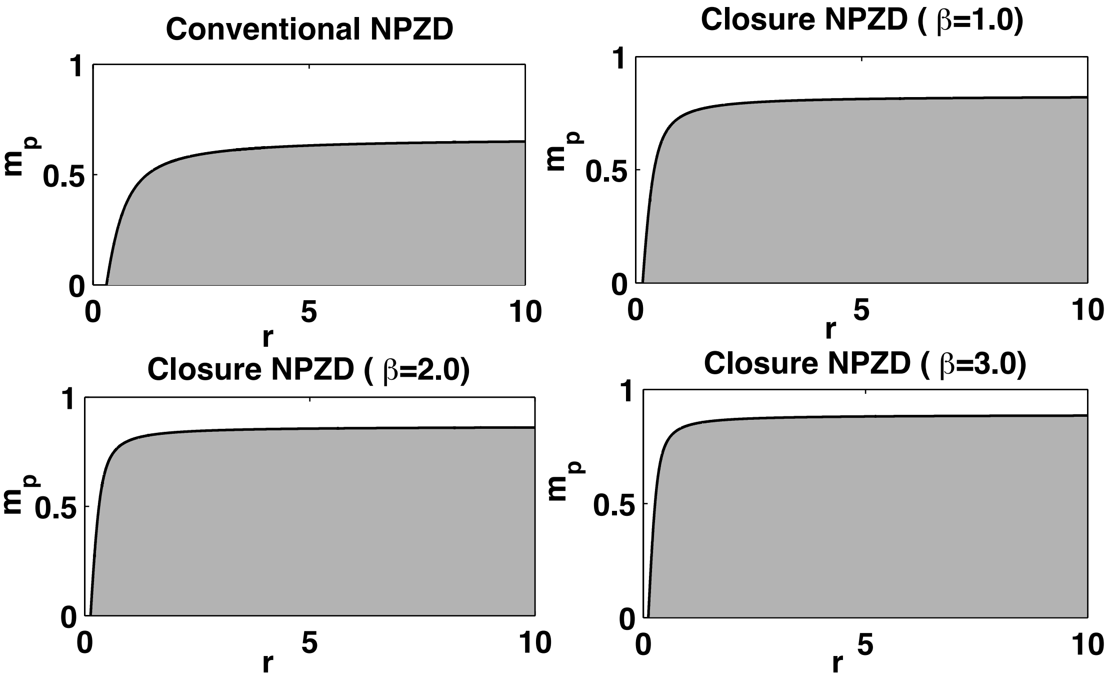

Fig. S10. Two-parameter (r, m_p) bifurcation diagram showing the parameter region for stable solutions of *N, P, Z* and *D* the NPZD model at different β.

Holling type-II Z-grazing function

Of all the empirical grazing response functions considered, the Holling type-II produces the widest range of CV_p, and only this grazing function produces periodic solutions, shown by the dark shaded regions in Fig. S11 for NPZD and in Fig. S12 for NPZ models. Again, the stability domain increases with β and is wider than for the conventional model.

Using the Holling type II grazing rate, the coefficient of variation CV_p may be less than 1 at low variability β while at high variability $CV_p>1$ (Fig. S14). In the field observations, CV_p varies from less than 1 to greater than 1 (Fig. 2, main text). With the linear Z-grazing function and the Holling type-III Z-grazing function, no stable solutions were obtained with $CV_p<1$.

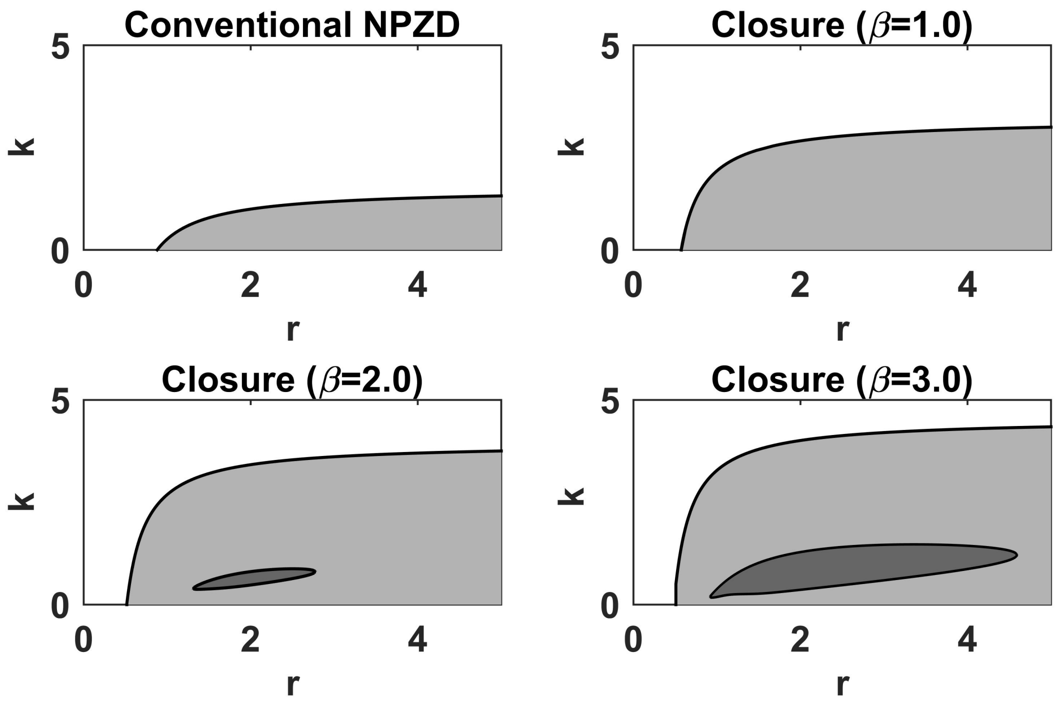

Fig. S11. Two-parameter (*r, k*) bifurcation diagram showing the parameter region (shaded area) for stable solutions of *N, P, Z* and *D* coexistence for the conventional NPZD model and the NPZD closure model at different β, all using the Holling Type II grazing response function. Periodic solutions in the NPZD closure model are possible (Black shaded region) when using the Holling Type II functional response.

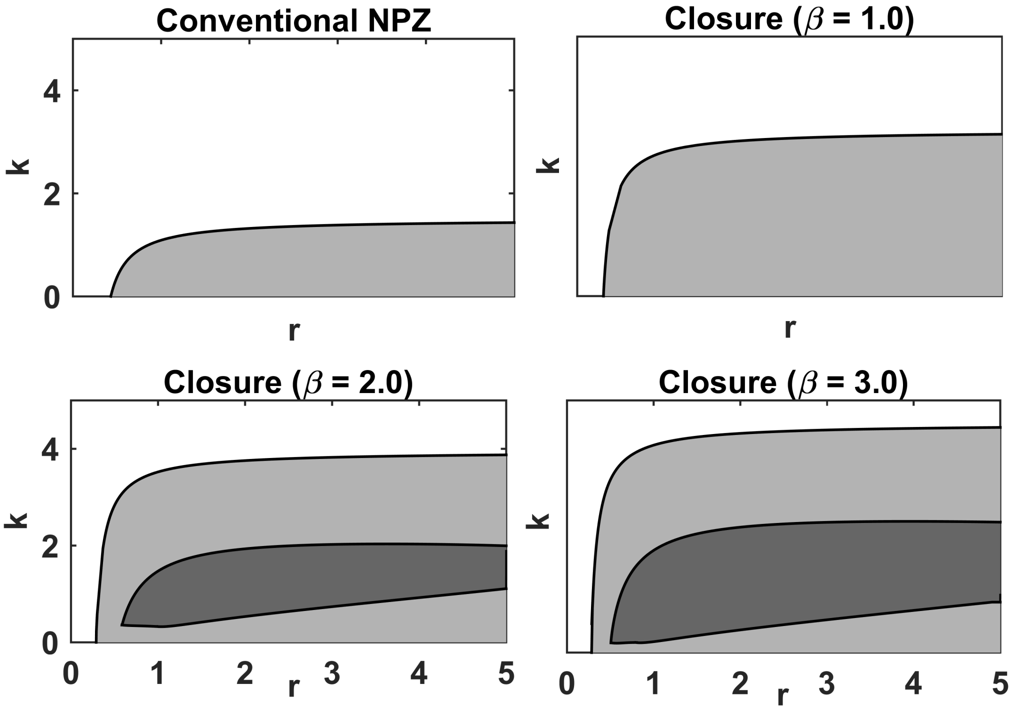

Fig. S12. Two-parameter (*r, k*) bifurcation diagram showing the parameter region for stable solutions of *N, P* and *Z* coexistence for the conventional NPZ model and the NPZ closure model at different β, all using the Holling Type II grazing response function. Periodic solutions in the NPZ model are possible (Black shaded region) when using the Holling Type II functional response.

Coefficient of variation (CV_P) of phytoplankton in the NPZD closure model

Fig. S13 shows the relationship between the CV_P and the micro-scale variability β for the NPZD closure model using the linear Z-grazing response. The diagrams are depicted using the parameter set (PAR) and with different values of the dimensionless phytoplankton mortality rate, m_p, which is directly related to $v_{\max}$ as $m_p=M_P/v_{\max}$. With the NPZD closure model a positive

correlation is obtained consistently between CV_P and β, and when using the linear (or Holing Type III) Z-grazing response all stable steady state solutions have $CV_P > 1$.

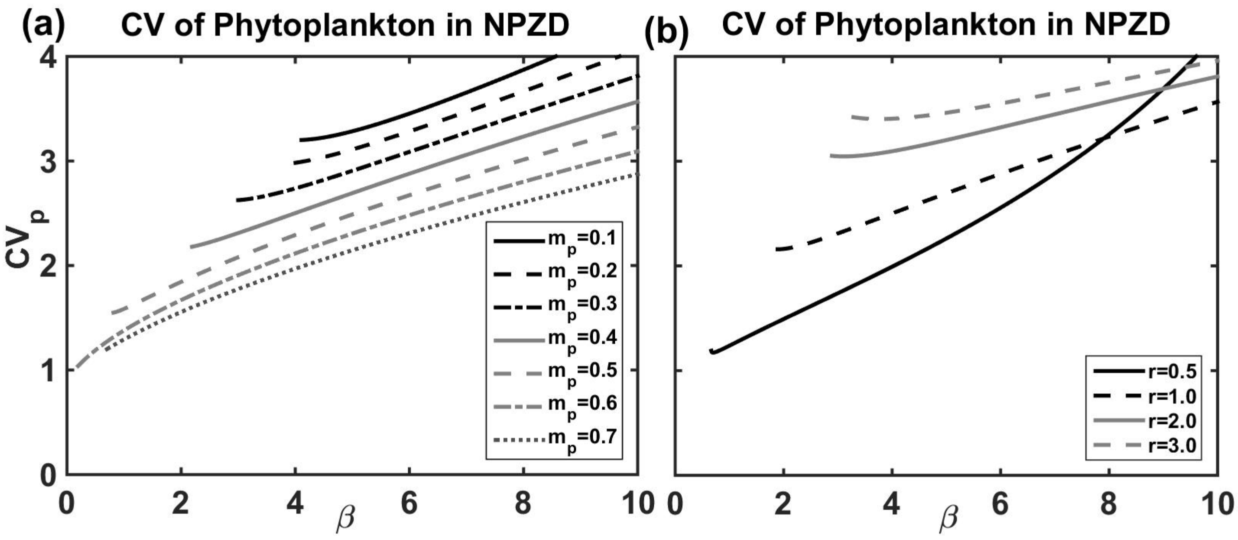

Fig. S13. a) Coefficient of variation of phytoplankton (CV_P) is shown with respect to standardized total variability β for different values of the dimensionless phytoplankton mortality rate, m_p= 0.1-0.7, and b) with respect to micro-scale variability β for different values of the dimensionless maximum grazing rate, r= 0.5-3.0. In both cases, CV_P increases with β and remains greater than one.

The coefficient of variation of phytoplankton, CV_P, is an important statistical quantity, which can be measured at millimetre scale resolution using TurboMap-L[1]. For the NP model and with non-saturating Z-grazing rate in the NPZ and NPZD models, all stable solutions have CV_P> 1. This result is robust as it is obtained consistently for NP, NPZ and NPZD interactions, with all combinations of parameter values that allow stable solutions. The observed coefficient of variation of phytoplankton is less than one for stations which are near the coast or within the

interior of Tokyo bay (Fig. 1 and 2, main text). Modelled CV_p< 1 is obtained only using the saturating Holling type-II Z-grazing rate, in either the NPZ or NPZD model (Figs. S15 and S16).

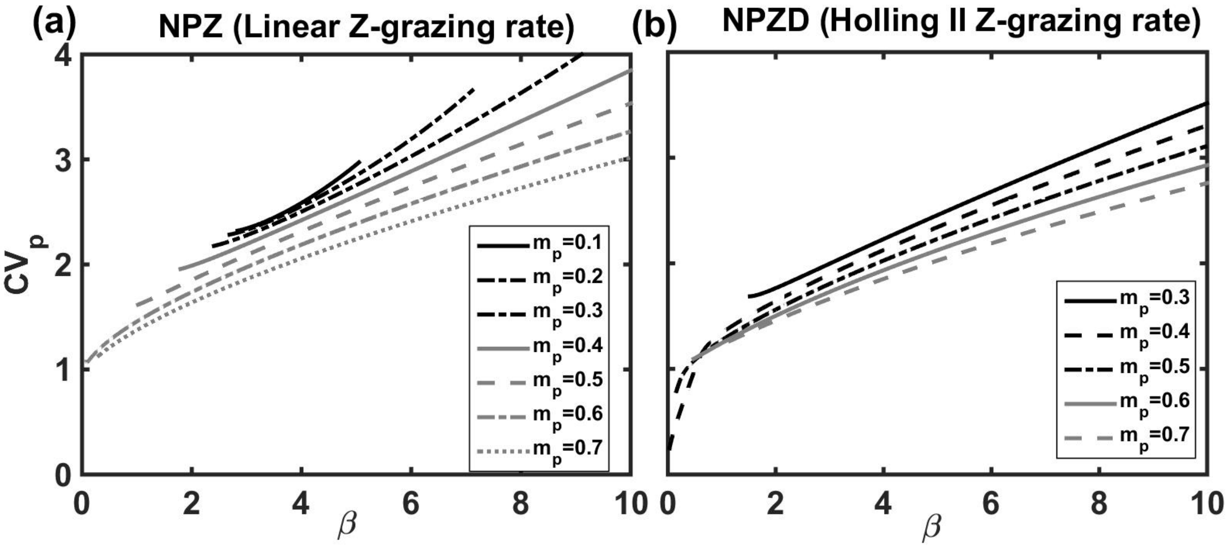

Fig. S14. Coefficient of variation against: a) the micro-scale variability, β in the NPZ Closure model with linear Z-grazing rate, and b) the micro-scale variability, β for different values of the mortality of phytoplankton m_p in the NPZ Closure model with Holling type II Z-grazing rate.

Trophic transfer efficiency and micro-scale variability

Modelled values of trophic transfer efficiency (*TE*) respond as expected to variations in model parameters. Both *TE* and CV_p increase with total micro-scale variability, β (Figs. S15 and S16). These two relationships are important because, together with the consistent positive relationships between CV_p and β (Figs. S13 and S14), they suggest that the observable CV_p can be both: 1) a good proxy for β, and 2) an index of *TE* in natural waters.

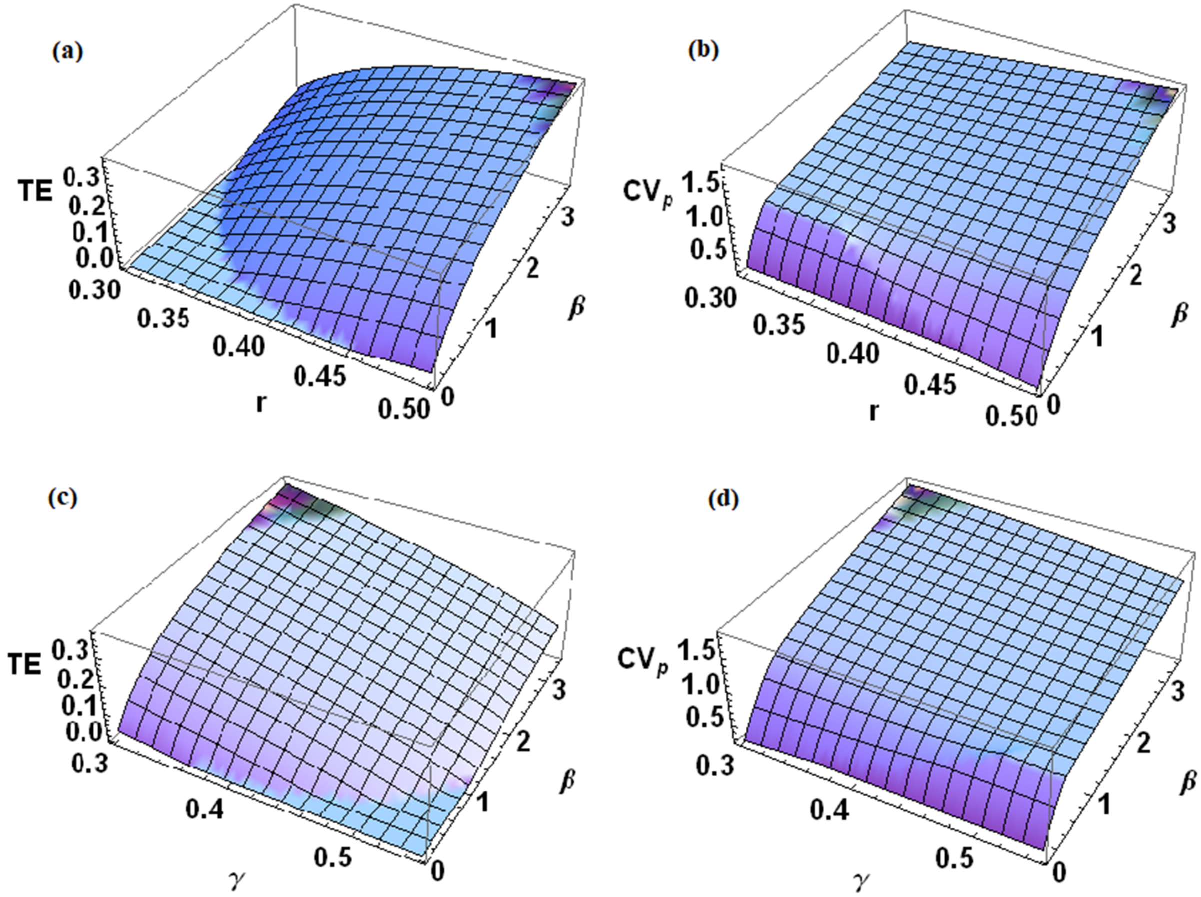

Fig. S15. *Coefficient of variation* CV_p of phytoplankton and *trophic transfer efficiency (TE)* from phytoplankton to zooplankton, as obtained with the NPZ closure model, shown versus micro-scale variability, β, and either: (a-b) maximum dimensionless z-grazing rate, *r*; (c-d) a fraction, γ of zooplankton grazing that becomes detritus. As expected, *TE* increases with maximum grazing rate and decreases with fraction, γ of zooplankton grazing that becomes detritus (with the remainder assimilated by zooplankton). Both *TE* and CV_p increase in a saturating manner with increasing micro-scale variability β.

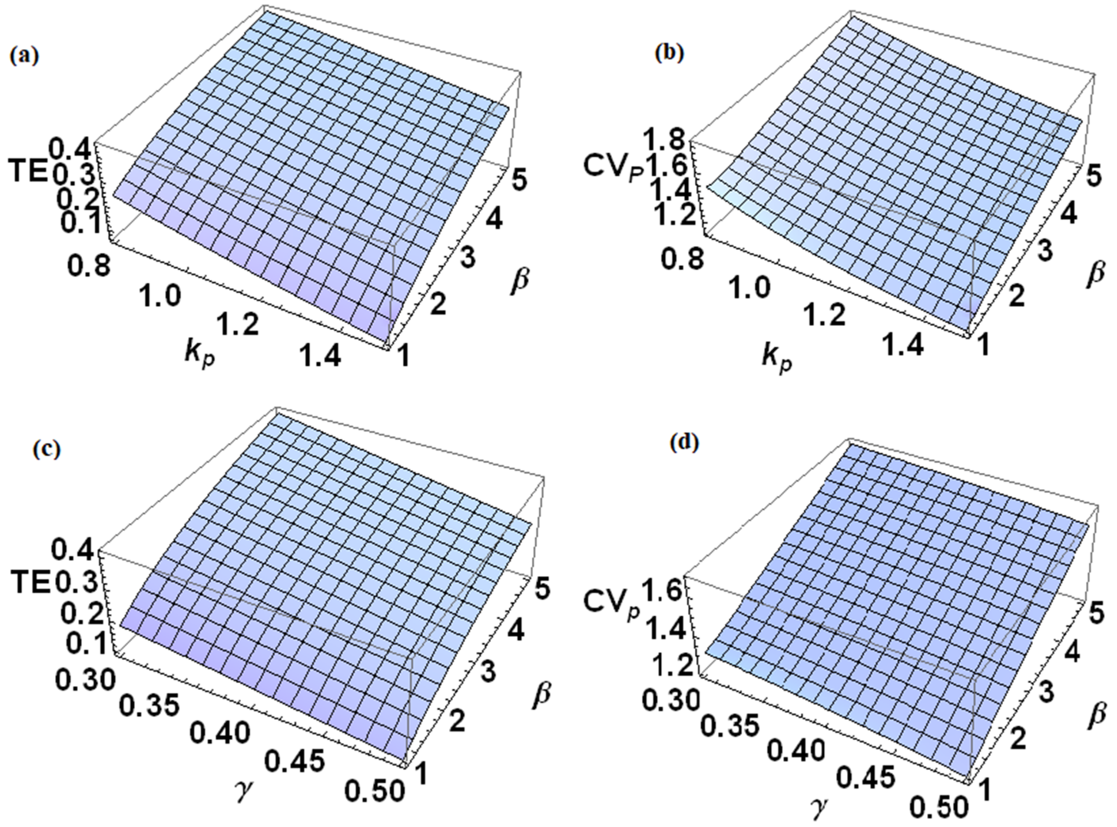

Fig. S16. Trophic transfer efficiency *(TE)* as obtained with the NPZD closure model, shown versus micro-scale variability, β, and either: (a) dimensionless half-saturation value for zooplankton grazing, k_p, or (c) fraction, γ of zooplankton grazing that becomes detritus (with the remainder assimilated by zooplankton). b) Modelled CV_p decreased with half-saturation value for zooplankton grazing, k_p, and d) with fraction, γ of zooplankton grazing that becomes detritus for range of micro-scale variability, β. Lower values of k_p, enhance grazing rate, and lower values of γ enhance assimilation of prey by zooplankton, either of which enhances *TE*. For the reasonable range of parameter values considered (Table S1), both *TE* and CV_p are impacted as much or more by changes in β as by variations in these model parameters.

References

1. Doubell, M. J., Yamazaki, H., Li, H. & Kokubu, Y. An advanced laser-based fluorescence microstructure profiler (TurboMAP-L) for measuring bio-physical coupling in aquatic systems. *J. Plank. Res.* **31**, 1441–1452 (2009).
2. Okada, T., Takao, T., Nakayama, K., Furukawa, K. Change in freshwater discharge and residence time of seawater in Tokyo Bay. *J. Jpn. Soc. Civ. Eng.* **63**, 67–72 (2007).
3. Uye, S. Replacement of large copepods by small ones with eutrophication of embayments: cause and consequence. (ed. Ferrari, F.D., Bradley, B.P.) *Ecology and Morphology of Copepods. Developments in Hydrobiology*. **102,** 513-519 (Springer, Dordrecht 1994).
4. Toyokawa, M., Furota, T., Terazaki, M. Life history and seasonal abundance of *Aurelia aurita* medusae in Tokyo Bay, Japan. *Plank. Biol. Ecol.* **47**, 48–58 (2000).
5. Barraquand, F. & Murrel, D. J. Scaling up predator-prey dynamics using spatial moments equations. *Methd. Ecol. Evol*. **4**, 276–289 (2013).
6. Stein, A., Gerstner, K. & Kreft, H. Environmental heterogeneity as a universal driver of species richness across taxa, biomes and spatial scales. *Ecol. Lett.* **17,** 866–880 (2014).
7. Wallhead, P. J., Martin, A. P. & Srokosz, M. A., Spatially implicit plankton population models: transient spatial variability. *J. Theo. Biol.* **253**, 405–423 (2008).
8. Doubell, M. J., Prairie, J. C. & Yamazaki, H. Millimeter scale profiles of chlorophyll fluorescence: Deciphering the microscale spatial structure of phytoplankton. *Deep-Sea Res II*. **101**, 207-215 (2014).

9. Foloni-Neto, H. et al. A new quasi-horizontal glider to measure biophysical microstructure. *J. Atmos. Oceanic Technol*. **31**, 2278–2293 (2014).

10. Mandal, S., Locke, C., Tanaka, M. & Yamazki, H. Observations and models of highly intermittent Phytoplankton distributions. *Plos ONE.* **9**, e94797 (2014).

11. Priyadarshi, A., Mandal, S., Smith, S. L. & Yamazaki, H. Micro-scale variability enhances trophic transfer and potentially sustains biodiversity in plankton ecosystems. *J. Theo. Biol.* **412**, 86-93 (2017).

12. Franks, P. J. S, Wroblewski, J. S. & Flierl, G. R. Behavior of a simple plankton model with food-level acclimation by herbivores. *Marine Biol.* **91**,121-129 (1986).

13. Clark, D. R. & Flynn, K. J. The relationship between the dissolved inorganic carbon concentration and growth rate in marine phytoplankton. *Proc. R. Soc. B.* **267,** 953-959 (2000).

14. Eppely, R.W., Rogers, J. N. & McCarthy, J. J. Half-saturation constants for the uptake of nitrate and ammonia by marine phytoplankton. *Limnol. Oceanogr.* **14**, 912-920 (1969).

15. Lehman, J. T., Botkin, D. B. & Likens, G.F. The assumptions and rationales of a computer model of phytoplankton production dynamics. *Limnol. Oceanogr.* **20,** 343-364 (1975).

16. Edward, A. M. & Brindley, J. Oscillatory behaviour in a three-component plankton population model. *Dyn. Stabil. Syst.* **11**, 347-370 (1996).

17. Cyr, H. Grazing of cladoceran- and copepod-dominated zooplankton communities in oligotrophic lakes. *Can. J. Fish. and Aq. Sci.* **55**, 414–422 (1998).

18. Sprules, W. G. Zoogeographic patterns in the size structure of zooplankton communities, with possible applications to lake ecosystem modelling and

management. (ed. Kerfoot, W. C.). *Evolution and ecology of zooplankton communities,* 642-656 (University Press of England, Hanover 1980).

19. Bertilsson, S., Hansson, L. A., Graneli, W. & Philibert, A. Size-selective predation on pelagic microorganisms in Arctic freshwaters. *J. Plank. Res.* **25**, 621–631 (2003).

20. Franks, P. J. S. NPZ models of plankton dynamics: their construction, coupling to physics, and application. *J. Oceanogr.* **58**, 379-387 (2002).

21. Fasham, M. J. R. Modelling the marine biota. (ed. Heimann, M.) *The global carbon cycle. 457-504 (*Berlin: Springer-Verlag 1993).

22. Dankowicz, H., Schilder, F. An extended continuation problem for bifurcation analysis in the presence of constraints, *J. Compt. Nonl. Dyn.* **6**, 031003; 10.115/1.4002684 (2011).
